# CovR and VicRK Regulate Cell Surface Biogenesis Genes Required for Biofilm Formation in *Streptococcus mutans*

**DOI:** 10.1371/journal.pone.0058271

**Published:** 2013-03-12

**Authors:** Rafael N. Stipp, Heike Boisvert, Daniel J. Smith, José F. Höfling, Margaret J. Duncan, Renata O. Mattos-Graner

**Affiliations:** 1 Department of Oral Diagnosis, Piracicaba Dental School, University of Campinas - UNICAMP, Sao Paolo, Brazil; 2 Department of Microbiology, The Forsyth Institute, Cambridge, Massachusetts, United States of America; 3 Department of Immunology and Infectious Diseases, The Forsyth Institute, Cambridge, Massachusetts, United States of America; Institut Pasteur, France

## Abstract

The two-component system VicRK and the orphan regulator CovR of *Streptococcus mutans* co-regulate a group of virulence genes associated with the synthesis of and interaction with extracellular polysaccharides of the biofilm matrix. Knockout mutants of v*icK* and *covR* display abnormal cell division and morphology phenotypes, although the gene function defects involved are as yet unknown. Using transcriptomic comparisons between parent strain UA159 with *vicK* (UAvic) or *covR* (UAcov) deletion mutants together with electrophoretic motility shift assays (EMSA), we identified genes directly regulated by both VicR and CovR with putative functions in cell wall/surface biogenesis, including *gbpB, wapE, smaA, SMU.2146c*, and *lysM.* Deletion mutants of genes regulated by VicR and CovR (*wapE, lysM, smaA*), or regulated only by VicR (*SMU.2146c*) or CovR (*epsC*) promoted significant alterations in biofilm initiation, including increased fragility, defects in microcolony formation, and atypical cell morphology and/or chaining. Significant reductions in mureinolytic activity and/or increases in DNA release during growth were observed in knockout mutants of *smaA*, *wapE, lysM*, SMU.*2146c* and *epsC*, implying roles in cell wall biogenesis. *WapE* and *lysM* mutations also affected cell hydrophobicity and sensitivity to osmotic or oxidative stress. Finally, *vicR, covR* and VicRK/CovR-targets (*gbpB, wapE, smaA*, *SMU.2146c, lysM, epsC*) are up-regulated in UA159 during biofilm initiation, in a sucrose-dependent manner. These data support a model in which VicRK and CovR coordinate cell division and surface biogenesis with the extracellular synthesis of polysaccharides, a process apparently required for formation of structurally stable biofilms in the presence of sucrose.

## Introduction

*Streptococcus mutans* is the major pathogen of dental caries and is commonly involved in bacteremia leading to infectious endocarditis [1]–[3]. The virulence of *S. mutans* lies in its ability to sense and adapt to environmental stresses during host colonization and biofilm formation (reviewed in [4]). This process involves systems for gene regulation designated two-component systems (TCS) [5]. A typical TCS comprises a histidine kinase (HK) membrane receptor which undergoes auto-phosphorylation in response to an environmental signal. The P-HK then transphosphorylates a cognate intracellular response regulator (RR), which in turn interacts with the regulatory regions of target genes. Cross-talk among TCS and with other regulatory systems also occurs [6]. The genome of *S. mutans* strain UA159 encodes 14 complete TCS [7], [8], including the conserved TCS of *Firmicutes* designated VicRK (Vic, for Virulence control), also known as WalkR/K or Yyc/FG, which controls cell wall metabolism in several Gram-positive species [9], [10]. Additionally, CovR (control of virulence; formerly named *gcrR*), is an orphan RR in the UA159 genome, since *covS*, the gene encoding the cognate HK, has not been identified in *S. mutans*, although it is present in other streptococcal species [7], [11]. In *S. mutans*, VicR and CovR directly regulate a panel of genes implicated in the synthesis of and interaction with extracellular polysaccharides [12]–[14], which are major components of the matrix of dental biofilms and essential for virulence (reviewed by [15]). Most of these genes are positively regulated by VicR [14], [16], and/or repressed by CovR (*gtfB/C/D*, *gbpC*). Glucosyltransferases B, C, D (encoded by *gtfB/C/D*) catalyze the synthesis of glucan polysaccharides from sucrose. Glucan-binding proteins B (GbpB) and C (encoded by *gbpB* and *gbpC* respectively) have affinity for glucan and are implicated in biofilm formation, cell wall integrity, and virulence by mechanisms not fully understood (reviewed in [17]). Phenotypes of *S. mutans covR* knockout mutants include abnormal biofilm structure, cell aggregation and attenuated cariogenicity [18], while *vicK* mutants show defects in separation of daughter cells, and in sucrose-dependent biofilm formation [14]. The altered functions associated with *covR*/*vicK*-mutant phenotypes warrant further investigation [16]. In this study, we analyzed the transcriptomes of *vicK* and *covR* mutants to identify new gene targets implicated in cell wall/envelope biogenesis and biofilm growth. Gene expression analyses and phenotypic characterization of knockout mutants of these genes indicate that CovR and VicRK regulate a set of genes implicated in cell wall biogenesis which are specifically activated during growth in the biofilm phase.

## Materials and Methods

### Strains, Plasmids, Growth Conditions and Reagents

Bacterial strains and plasmids used in this study are shown in Table 1. *Esch*e*richia coli* was grown aerobically in Luria-Bertani medium supplemented as needed with ampicillin (100 µg/ml), erythromycin (200 µg/ml) or spectinomycin (200 µg/ml). *Streptococcus mutans* strains were routinely grown in Brain Heart Infusion (BHI) or Todd Hewit Broth (THB) as described previously [19]; when necessary erythromycin (erm, 10 µg/ml) and/or spectinomycin (spec, 300 µg/ml) were added to the media. Growth curves of the studied strains were performed as previously described [7], with minor modifications. PCR primers are shown in Table S1.

**Table 1:** Strains and plasmids used in this study.

Strains and plasmids	Relevant characteristics or purpose	Source
UA159	Erm^s^, spec^s^	ATCC
UAcov	Δ*covR*::Erm^r^	[20]
UAvic	Δ*vicK*::Erm^r^	[16]
UAwapE	Δ*wapE*::Erm^r^	This study
UAlysM	Δ*lysM*::Erm^r^	This study
UA2146c	Δ*SMU.2146c:*:Erm^r^	This study
UAsmaA	Δ*smaA*::Erm^r^	This study
UAepsC	Δ*epsC:*:Erm^r^	This study
UA159-pdl	UA159/pDL278; Spec^r^	This study
UA159-pva	UA159/pVA838; Erm^r^	This study
UAcov+	Δ*covR*::Erm^r^; pDL278::*SMU.1924;* Spec^r^	[20]
UAvic+	Δ*vicK*::Erm^r^; pDL278::*SMU.1516*; Spec^r^	[16]
UAwapE+	Δ*wapE*::Erm^r^; pDL278::*SMU.1091;* Spec^r^	This study
UAlysM+	Δ*lysM*::Erm^r^; pDL278::*SMU.2147c*; Spec^r^	This study
UA2146c+	Δ*SMU.2146c:*:Erm^r^; pDL278::*SMU.2146c*; Spec^r^	This study
UAsmaA+	Δ*smaA*::Erm^r^; pDL278::*SMU.609*; Spec^r^	This study
UAepsC+	Δ*epsC:*:Erm^r^; pDL278::*SMU.1437c*; Spec^r^	This study
pVA838	(erm^r^ ) source	[56]
pDL278	(Spec^r^) cassette; low-copy number vector for construct of complemented strains	[57]
pET22B	(amp^r^) Empty vector for construct and expression of His-Tag proteins	Novagen
*E. coli* DH5-α	General cloning and plasmid amplification	Invitrogen
*E. coli* BL21	Expression of pET22B::*covR* and pET22B::*vicR*	Novagen

### Construction of Knockout Mutants

Knockout strains were constructed by PCR ligation mutagenesis as described elsewhere [16], [20] with minor modifications. Amplicons were generated with Taq DNA Polymerase High Fidelity (Invitrogen) from genomic DNA templates. The erythromycin resistance gene was amplified from plasmid pVA838. Amplicons obtained with primers E1/E2 and primer set P1/P2 for each gene were digested with AscI, purified using the StrataPrep Purification Kit (Stratagene), and ligated with T4 DNA ligase, according to the manufacturer’s protocol. Amplicons obtained with primers E1/E2 and respective primer set P3/P4 for each gene were digested with XhoI, purified and ligated. Ligation products were purified and used as templates in a PCR reaction with primers P1/P4, yielding mutant alleles in which target genes were disrupted by an erythromycin resistance cassette (ERM). Purified ERM-inactivated mutant alleles were transformed into *S. mutans* UA159 and transformants were confirmed as described previously [16]. To generate complemented strains, each mutant was transformed with plasmid pDL278 containing the intact copy of the respective deleted gene.

### Biomass Quantification of Biofilms

To measure mature biofilm biomass formed during growth in media with sucrose, quantitative biofilm assays in polystyrene 96-well microtiter plates (round-bottom) were performed as previously described [21] with some modifications. Briefly, 500 µl of a BHI culture (A_550 nm_ 0.3) was transferred to 4.5 ml of fresh medium containing 0.1% sucrose. Aliquots (250 µl) were transferred in triplicate to polystyrene 96-well plates (BD Biosciences) and incubated in 10% CO_2_ for 8 and 18 h. After three washes with distilled water to remove planktonic or loosely attached cells, biofilms were stained with crystal violet for 30 min. Stain was eluted from biofilms with ethanol (30 min incubation), and absorbances of eluates were measured at A_575 nm_ and expressed as amounts of biofilm biomass. Absorbances of cell suspensions (A_550 nm_) from each culture were measured as a control for planktonic growth. Similar assays were performed using Chemically Defined Medium [22], [23] supplemented with 0.1% sucrose and Brucella broth supplemented with 5% blood and 0.1% sucrose. Amounts of biofilm biomass were expressed as means of three independent experiments performed in triplicate (2, 4, 18 h-biofilms) or six replicates (8 h-biofilms).

### Electron Microscopic Analysis of Strains during Planktonic and Initial Phases of Biofilm Growth

The morphologies of UA159, knockout, and respective complemented strains were analyzed at mid-log phase of planktonic growth in the presence or absence of sucrose, and during initial phases of sucrose-dependent biofilm formation by scanning electron microscopy (SEM) as described elsewhere [16]. Briefly, overnight cultures of each strain were 100-fold diluted in fresh media and incubated until optical density (A_550 nm_) reached 0.3. Planktonic cells were harvested by centrifugation, washed three times with PBS and processed in microtubes for SEM analysis. To analyze cells in biofilms, 1.0 ml of cultures (A_550 nm_ 0.3) were transferred to 24-well plate containing sterile glass slides. Plates were gently mixed and incubated for 2 and 4 h for biofilm formation. Biofilms were washed three times with PBS to remove non-adherent cells and processed for SEM analysis, as described previously [16]. Quantitative analyses of biofilm phenotypes used SEM digital images at 1,300× magnification of 32 pre-determined areas (97 to 63 µm) of each sample equally distributed on each glass slide. Numbers of microcolonies and total covered surfaces per area were determined using ImageJ – Image Processing and Analysis software in Java (NIH, http://rsbweb.nih.gov/ij/index.html). Parametric analysis of variance (ANOVA) with *post hoc* Dunnett’s test was used to compare number of microcolonies and total covered areas between strains.

### RNA Purification, Protein Extraction, Western and Northern Blotting

For RNA or protein extraction, cultures in BHI (A_550 nm_ 0.3) were harvested by centrifugation (6000×g, 4°C, 3 min), washed twice in cold saline, suspended in 220 µl 10 mM Tris, 1 mM EDTA, pH 8.0 (TE), and stored at −80°C until use. Mechanical disruption of cells was carried out with 0.16 g 0.1 mm diameter Zirconium Beads (Biospec) on a Mini-beadbeater (Biospec) at maximum power. For RNA isolation a modified protocol from the RNeasy Mini Kit (Qiagen) was used. Briefly, cells were subjected to bead beating (3 cycles of 30 sec with 1 min rest on ice), RLT buffer (850 µl) was added and the suspension homogenized by vortexing. After centrifugation (10,000×g, 1 min, 4°C), supernatants (700 µl) were mixed with ethanol (500 µl) and loaded onto columns. Further RNA purification was performed as recommended by the manufacturer. To remove chromosomal DNA, samples were treated with 10 U Turbo DNase (Ambion) according to the manufacturer’s protocol. For whole cell protein extraction, frozen cell pellets were suspended in 350 µl water followed by bead beating (3 cycles of 60 sec with 1 min rest on ice). Extracts were briefly centrifuged (7,000×g, 30 sec) and supernatants containing the whole cell protein fraction were stored at −80°C until use. Protein concentration was measured by Bradford assay (BioRad) according to the manufacturer’s protocol.

For western blot assays, equal amounts of protein from whole cells (0.5 µg) or cell-free supernatants (2.5 µg) of cultures (A_550 nm_ 0.3) were resolved in 10% SDS-PAGE gels and electro-transferred to PVDF membranes. The amounts of protein used were within a linear range for GbpB detection, as determined in western blots with a standard curve of purified GbpB. For determination of protein concentration in culture supernatants, samples were dialyzed against PBS pH 7.4 then 100-fold concentrated by lyophilization. Membranes were incubated with rat GbpB-specific anti-serum [16], rabbit CovR-specific anti-serum or VicR-specific anti-serum (this study) at 1∶1,000 or 1∶500 dilutions. After probing with goat anti-Rat IgG or goat anti-Rabbit IgG, secondary antibodies conjugated with horse-radish peroxidase (dilution 1∶10,000); signals were detected with the Pierce ECL Substrate (Pierce). Autographs were scanned in a BioRad GS-700 Imaging Densitometer and values of band intensities were expressed as arbitrary units to estimate amounts of GbpB, VicR or CovR. CovR and VicR were not detected in culture supernatants of *S. mutans* strains (data not shown).

Northern blot assays were performed as previously described [23] with some modifications. Briefly, equal amounts (4 µg) of total RNA purified from mid-log phase of growth (A_550 nm_ 0.3) of UA159 after 0, 2.5, 5, 10, 15, 20 and 30 min of culture exposure to 300 µg/ml of rifampin (Sigma). Samples were resolved by electrophoresis in 1% formaldehyde-agarose gels and transferred to positively charged Nylon membranes (Amersham). Probes were obtained in PCR reactions with primer sets gbpB-qPCR and gbpC-qPCR, using UA159 chromosomal DNA as template. PCR products were column-purified (QIAquick PCR Purification Kit, Qiagen) and labeled using the DIG Luminescent System (Roche). Blots were incubated with 10 ng/ml probes at 58°C, and signals were detected using the CSPD Star substrate (Roche) as recommended by the manufacturer. Autographs were analyzed as described for western blotting.

### Analysis of the Transcriptomes of *vicK* and *covR* Mutants

Comparative transcriptome analyses of UA159 and mutants UAvic and UAcov were carried out using *S. mutans* UA159 microarrays provided by the Comprehensive Microbial Resource (CMR) of the J. Craig Venter Institute. Direct regulation of *gtfB/C* and *gbpC/B* genes by CovR and/or VicR were shown to occur at mid-log and stationary growth phase [12], [14], [16], therefore, cells were harvested at mid-log phase of growth in BHI (A_550 nm_ 0.3) when expression of *vicR* and *covR* in several *S. mutans* strains, including UA159, is maximal [19]. Experiments were carried out using the CMR protocol M007 (revision 2.0) and M008 (revision 2.1). Briefly, cDNA was generated with random primers from 2 µg total RNA using Superscript III RT (Invitrogen), and conjugated with either Cy3 or Cy5 dyes. Slides were hybridized, washed, and scanned using a Genepix 4000B scanner (Axon Molecular Devices). Fluorescence intensities were quantified using GenePix Pro 6.0 software (Axon). The resulting files (*.gpr) were analyzed with the LIMMA algorithm interface available online at www.brop.org. A p value <0.001 confidence level and PDE (probability of differential expression) >50% were used to pinpoint significantly differentiated genes. In accordance with MIAME standards [24], the microarray data for UAvic and UAcov are available online at URL www.brop.org/idn:12496045393872 and www.brop.org/idn:12496187486126, respectively. Transcriptional activities of genes identified in the microarray analysis were confirmed by RT-qPCR.

### Reverse Transcription and Quantitative PCR

Reverse transcription was carried out with 1 µg RNA using Superscript III RT (Invitrogen) as previously described [19]. Quantitative PCR was performed in an iCycler System (Biorad), and the reaction mix included template cDNA (30 ng), 30 µM of each primer, and 1× SYBR-Green mix (Biorad). The thermal cycling conditions were: 95°C for 3 min for the initial denaturation, followed by 45 cycles of three steps consisting of denaturation at 94°C for 15 s, primer annealing at 54°C for 15 s, and primer extension at 72°C for 30 s. A standard amplification curve and a melting-point product curve were obtained for each primer set. The expression levels of the genes tested were normalized relative to the expression of the 16S rRNA gene of *S. mutans* [19]. Statistical analysis was performed using one-way analysis of variance (ANOVA) with *post hoc* Dunnett’s test. Differences were considered significant at p-values lower than 0.05. Assays were performed in duplicate with at least three independent RNA samples.

### Production of Recombinant CovR, VicR, and Polyclonal Antibodies

To generate CovR- and VicR-His-Tag fusion proteins, each ORF was amplified with primers covRHisF and covRHisR or vicRHisF and vicRHisR (Table 1). PCR amplicons were restricted with NcoI and XhoI and purified products were cloned into NcoI-XhoI-digested pET-22b (Novagen) to yield pET-covR or pET-vicR. Plasmids were transformed into *E.coli* BL21, and recombinant proteins were isolated from 1 l cultures (A_550 nm_ 0.8) after 3 h induction with 1 mM IPTG. After cell lysis, recombinant proteins were purified by affinity chromatography on Ni^2+^ NTA agarose (Qiagen) and eluted r-proteins were dialyzed overnight in phosphate buffered saline (PBS) at 4°C. Aliquots of purified proteins were stored at −20°C and purity/integrity was visualized by Coomassie staining after SDS-PAGE. Polyclonal antibodies to r-CovR and r-VicR were produced using the standard 77 Days Rabbit Protocol (Covance). Antibody specificity to r-proteins was evaluated by Western blot and ELISA assays.

### Electrophoretic Mobility Shift Assays (EMSA)

To investigate if genes identified in transcriptional analyses of UAvic and UAcov were directly regulated by VicR and/or CovR, binding of the regulators to gene promoter regions was analyzed in EMSA assays. PCR amplicons of each candidate promoter region were generated, purified and end-labeled with the DIG Gel Shift Kit (Roche). For phosphorylation, r-CovR or -VicR was incubated (45 min, 25°C) with 50 mM acetyl phosphate prior to the binding reaction [13]. Binding reactions with r-CovR were carried out in 25 µl volumes containing 1× Roche DIG Gel Shift Kit Buffer (20 mM Hepes, 1 mM EDTA, 10 mM (NH_4_)_2_SO_4_, 1 mM DTT, 0.2% Tween 20, 30 mM KCl, pH 7.6), with poly L-lysine (5 ng/µl), unspecific competitor Poly [d(A-T)] (50 ng/µl), labeled target DNAs (∼0.6 fmoles) and increasing amounts of unphosphorylated or phosphorylated r-CovR (0, 7.5, 15, 22.5 and 30 pmoles). Binding reactions with r-VicR were carried out in 25 µl volumes containing reaction buffer (52.5 mM MOPS, 9.5% glycerol, 50 µM EDTA, 50 µg/ml BSA, pH 7.4), 50 ng salmon sperm DNA as unspecific competitor, labeled target DNAs (∼0.6 fmoles) and increasing amounts of unphosphorylated or phosphorylated r-VicR (0, 30, 60, 90 and 120 pmoles), as previously described [15].

The range of r-CovR and r-VicR concentrations used in these assays were previously identified [13], [15] and confirmed to be suitable for specific binding to the *gtfB* promoter region (−159 to +36) in preliminary experiments. Samples were incubated (60 min, 25°C) and protein-DNA complexes were separated on 6% DNA Retardation acrylamide gels (Invitrogen) at 70 V for 3 h. Protein-DNA complexes were visualized in gels using the Electrophoretic Mobility-Shift Assay Kit (Molecular Probes, Life Tech) or detected using DIG wash and block buffers after electro-transfer to positively charged nylon membranes (Roche), according to the respective manufacturer’s protocol.

Specificity of binding in each assay was guaranteed by competition with 120-fold or 200-fold (for VicR) excess of respective un-labeled probe (cold-DNA). All promoter region fragments were approximately the same length (≈300 bp). Of note, co-binding assays were performed in the reaction buffer described for r-VicR, in which r-CovR showed the same binding capacity.

### Cell Hydrophobicity Assays

Cell surface hydrophobicity was determined as described elsewhere [25]. Briefly, cells from BHI cultures (A_550 nm_ 0.3) were harvested (10,000×g, 5 min), washed twice with PUM buffer (22.2 g K_2_HPO_4_•3H_2_O, 7.26 g KH_2_PO_4_, 1.8 g urea, 0.2 g MgSO_4_•7H_2_O per liter, pH 7.1) and resuspended in the same buffer at optical density A_550 nm_ 0.900. Suspensions (3 ml) were mixed with 400 µl of hexadecane, by vortexing twice for 30 s, and incubated at 30°C for 30 min. Optical densities of the aqueous phases were measured at A_550 nm_ and hydrophobicity indexes were calculated in relation to A_550 nm_ of respective suspensions without hexadecane. Three independent experiments were performed in triplicate.

### Mureinolytic and Autolysis Assays

The ability to cleave β-(1,4)-linkages between N-acetyl-muramic acid and N-acetyl-D-glucosamine of murein, was determined in UA159, knockout and respective complemented strains, using the EnzChek Lysozyme Assay Kit (Molecular Probes, Invitrogen). Briefly, cells were grown to mid-log phase and 50 µl aliquots of cultures were tested for lytic activity on *Micrococcus lysodeikticus* cell walls, which are fluorescently labeled and quenched. After 15 min incubation in the dark, 37°C, fluorescence of cleaved products was measured in a microplate reader using emission/excitation of 485/530 nm according to the manufacturer’s protocol. A standard curve generated with lysozyme chicken egg white was used to calculate activities of bacterial samples. Activities were corrected for the A_550_ of cultures at the time of assay and expressed relative to UA159 activity which was set to 1.0. Assays were performed in triplicate in at least three independent experiments.

The autolytic activities of strains were analyzed using a previously described assay [22]. Planktonic cells were centrifuged (10,000×g, 5 min) and washed twice with PBS. Cell pellets were re-suspended in phosphate buffer (20 mM, pH 6.5 with 1 M KCl, 1 mM CaCl_2_, 1 mM MgCl_2_ and 0.4% sodium azide) to A_550 nm_ 0.9. Cell suspensions were incubated at 44°C and autolysis was monitored spectrophotometrically (A_550 nm_) at 24, 48 and 72 h. At least three independent experiments were performed for each strain.

### Quantification of Extracellular DNA (eDNA) in Biofilm Supernatants

The release of DNA during biofilm formation was measured in UA159, mutant and complemented strains, as described previously [26] with some modifications. Culture supernatants from 8 h biofilms were collected and harvested by centrifugation (twice at 13,000×g, 5 min, 4°C). Volumes of the cell-free samples (2 µl) were added to 1× SYBR® Green PCR Master Mix (Life Tech) containing primers for 16S RNA gene (Table S1). DNA amounts were determined by qPCR and calculated from four independent experiments and expressed relative to that from UA159 biofilms (set as 1.0).

### Viability Under Osmotic and Oxidative Stress

Analysis of strain sensitivities to osmotic and oxidative stress was performed as previously described [16]. Briefly, cultures were grown to mid-log phase (A_550 nm_ 0.3) and exposed to osmotic stress (5 M NaCl for 30 min) or to oxidative stress (10 µM H_2_O_2_ for 1 h, followed by 100 µM H_2_O_2_ for 30 min). After stress exposure, serial dilutions of cultures were plated on BHI agar to determine the number of viable cells. Similar cultures not exposed to stress were used as control. Three independent experiments were performed in triplicate.

### *In silico* Analysis of Promoter Sequences and Functional Classes of Genes

Promoter sequences were retrieved from the Oralgen Database, Los Alamos National Laboratories (www.oralgen.lanl.gov) and screened to identify VicR and CovR binding motifs. Promoter screening was performed using computational programs (Gibbs MotifSampler http://www.bayesweb.wadsworth.org/gibbs/gibbs.html, AlignACE http://www.atlas.med.harvard.edu and LALIGN http://www.ch.embnet.org/software/LALIGN_form.html programs). Putative functions and conserved domains of proteins encoded by genes identified in microarray screens were examined using the NCBI database (http://www.ncbi.nlm.nih.gov/gene), in which information from GeneRIF (Gene Reference into Function) and relevant publications when available, were analyzed. Functional domain(s) were searched using the Non-redundant protein sequence database (RefSeq) and the Conserved Domain Database at NCBI [27].

## Results

### Mutations in VicK and CovR Affect Expression of Genes with Putative Roles in Cell Wall or Cell Envelope Biogenesis

Microarray-based transcriptomic comparisons between UA159 parent and *vicK* and *covR* mutant strains (UAvic and UAcov, respectively) are shown in Table S2. The fold-expression changes detected by microarray either in individual genes or in the first and last genes of potential operons were confirmed by RT-qPCR and were statistically significant (Table S2, p<0.01–0.05). Using a conservative cut off of a 3-fold expression change, a total of 23 open-reading frames (ORFs) were down-regulated and 1 was up-regulated in mutant UAvic. Eleven of the down-regulated ORFs were organized into three operon-like clusters (SMU.1434c-1437c; SMU.1004–1005; SMU.1334c–1336c). Thus, a total of 15 loci were significantly down-regulated in this mutant (Table S2). Also included in Table S2 are four genes that showed changes which were slightly below the 3-fold cut-off in microarrays. These were analyzed by RT-qPCR because of previous evidence of direct regulation by VicR, e.g. *gtfB/C* [14] or their potential functions in cell wall biogenesis (*wapE* and *SMU.367*). Previous analyses of *gtfB/C* transcription in a UA159 *vicK* mutant did not reveal strong up-regulation of *gtfB/C*, although promoter regions of these genes directly interact with VicR [14]. Thus, a 3.0 and 1.4-fold down-regulation of *gtfB*/*C* observed in UAvic (Table S2), is comparable to previous observations [14].

Functional classification of gene products most significantly down-regulated in UAvic illustrates the major role of the VicRK TCS in regulating genes associated with the synthesis of and interaction with the extracellular matrix of biofilms and for cell wall biogenesis (Table S2). The most strongly down-regulated gene in UAvic was *SMU.2146c*, encoding a protein with a SLT transglycosylase domain (Soluble Lytic Transglycosylase), typical of lytic proteins which cleave murein linkages. This gene forms a cluster with *SMU.2147c*, which was designated *lysM* in this study, because it encodes a protein with a LysM lysin domain typical of autolysins. Additionally, the transcription data indicate that *smaA,* encoding an autolysin [28], is strongly repressed by VicRK (Table S2). Other genes strongly down-regulated in UAvic encode proteins involved in fatty acid biosynthesis, e.g. *SMU.1334c-SMU.1336c*. *GbpD* could also be included in this functional class of proteins, since in addition to a glucan-binding function, its participation in lipid metabolism was also established [29]. G*tfB, gbpB* and *comC* (encoding the competence peptide ComC) were also strongly down-regulated in UAvic. Down-regulation of *wapE* in UAvic was similar to that observed for *gtfC*, a gene shown to be directly regulated by VicR [14]. Of note, *vicR* and *vicX* (not detected in microarray) were 1.7-fold and 2.4-fold down-regulated in UAvic, respectively, as quantified by RT-qPCR. In addition, VicR down-regulation was confirmed at the protein level (data not shown).

In the UA159 *covR* mutant (UAcov), using a conservative cut off of 2.5-fold change, 29 ORFs were significantly up-regulated, of which 14 were organized into 4 operon-like loci. As anticipated, *gtfB* and *gpbC* were strongly up-regulated (Table S2). Given the results showing CovR binding to the *gbpB* promoter (below), RT-qPCR analysis of *gbpB* transcripts was also performed, which indicated a 1.4-fold increase in UAcov (Table S2, p<0.05). A total of 8 (27.8%) of the 29 genes up-regulated in UAcov encoded proteins with putative functions in cell wall biogenesis. These include *SMU.575c* (an exporter of murein hydrolases), *wapE*, *SMU.1434c* and *epsC* (*SMU.1437c*, from the operon-like locus *SMU.1437c-SMU.1434c*), *SMU.1918c* (from locus *SMU1918c-1923c*), and *lysM*. In addition, a total of 24 ORFs were significantly down-regulated in UAcov. These included 13 genes that were also down-regulated in the mutant UAvic (*SMU.1334–1335–1336–1338, SMU.1339–1341–1342–1344–1346*; Table S2) and 11 not significantly affected in UAvic (Table S3).

### CovR and VicR Directly Regulate Genes Involved in Cell Wall and Cell Envelope Biogenesis

**Figure 1:**
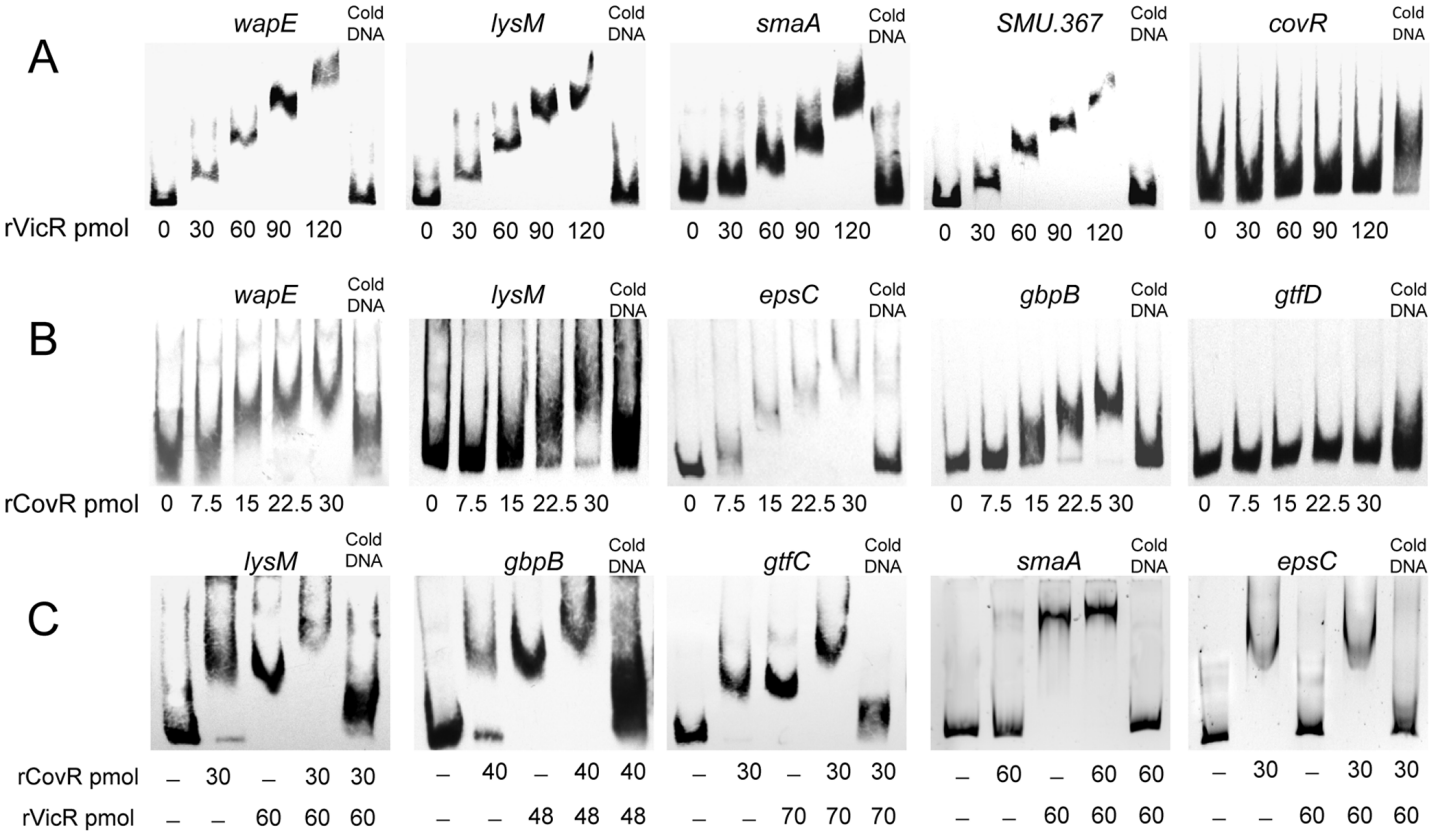
EMSA analysis with promoter regions of genes potentially regulated by VicR and/or CovR. Genes which showed significant fold-changes in expression in UAvic and/or UAcov mutants compared to UA159 were selected. (A) Recombinant VicR protein (rVicR) specifically bound to the promoter regions of *wapE*, *lysM*, *smaA* and *SMU.367*; *covR* was a negative control fragment of similar size. (B) rCovR specifically bound to the promoters of *wapE*, *lysM*, *epsC* and *gbpB*, but not to *gtfD*. (C) EMSA assays performed in the presence of the two regulators (rCovR and rVicR) showed co-binding to the promoter regions of *lysM*, *gbpB* and *gtfC*. Co-binding was not observed with the promoter fragments of *smaA* and *epsC*, which are exclusively regulated by VicR and CovR, respectively. Specificity of binding was confirmed in competitive assays with excess of cold DNA.

EMSA assays were performed with r-CovR and r-VicR, individually and in combination, to determine whether they interact directly with the promoter regions of genes identified by transcriptome analyses as potentially regulated by both VicR and CovR (i.e. *wapE, lysM*, and *gbpB*), by VicR only (*SMU.367* and *smaA*), and by CovR only (*epsC*). Negative controls for r-VicR and r-CovR interactions were *covR* and *gtfD,* respectively, since we established previously that promoters of these genes were not bound by these RR. VicR binds to the promoters of the newly identified genes (*SMU.367*, *wapE, smaA,* and *lysM)* (Fig. 1A). CovR could bind to promoters of *wapE*, *lysM,* and *epsC,* and *gbpB* (Fig. 1B). To investigate whether r-CovR and r-VicR co-bind the promoters of genes that were regulated by each RR individually, we selected *lysM*, *gbpB,* and *gtfC* (shown to be regulated by VicR and CovR) [12], [14], *epsC* (regulated only by CovR) and *smaA* (regulated only by VicR). CovR and VicR could co-bind to the promoters of *lysM, gbpB* and *gtfC* leading to increased retardation of the probes compared to control genes regulated by only one regulator (Fig. 1C).

Direct regulation of *gbpB* by CovR was further investigated by comparing the amounts of GbpB in cell extracts and in culture supernatants of UA159, UAcov and the complemented strain. The *vicK* mutant (UAvic) was used as a control, since GbpB production is depleted in this strain [16]. Compared to the parent, there was significantly higher production of GbpB in UAcov, which was restored to parental levels in the *covR* complemented strain (Fig. S1). The increase in GbpB expression in UAcov was 4-fold higher at the protein level (cell-associated and secreted GbpB), and 1.4-fold higher at the transcript level. Therefore, we compared the stability of *gbpB* and *gbpC* transcripts, by quantifying the amounts of transcript in equal amounts of RNA purified from UA159 (A_550 nm_ 0.3) at 0, 2.5, 5, 10, 20, 30 and 60 min after addition of rifampin to block RNA polymerase. These analyses revealed that *gbpB* transcripts have a half-life approximately 3-times longer compared to *gbpC* transcripts (Fig. S1).

**Figure 2:**
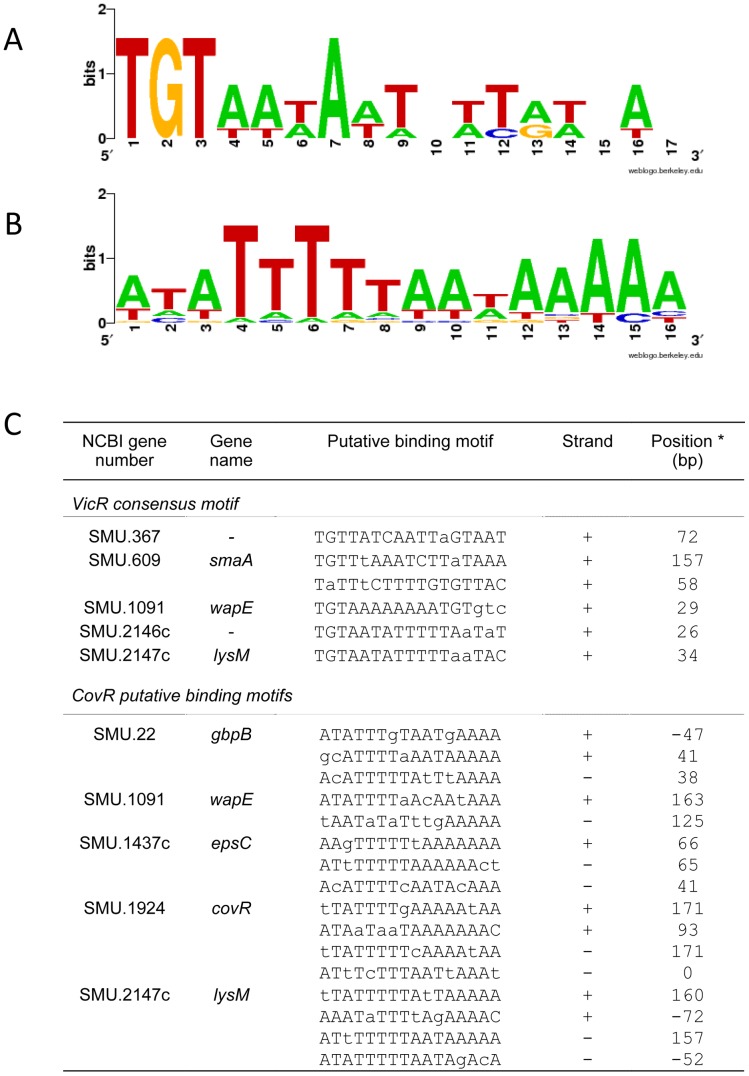
WebLogo representation of the position weight matrices derived from VicR or CovR regulated promoters. (A) VicR and (B) CovR consensus sequences. (C) Sequence and position of the VicR/CovR binding sites in each gene promoter. Consensus for VicR: TGTWAHNNNNNTGTWAH [9], and consensus for CovR AWATTTTTAAWAAAAR where W is A or T and R is C or A [30]. Lower case indicates mismatch. * Distance from putative translation start site.

The VicR consensus binding motif [9] was found in the promoter regions of all other genes involved in cell wall biogenesis including *SMU.367*, *smaA*, *wapE*, *lysM* and *SMU.2146c* (Fig. 2). The VicR binding sequence was also reported in the promoter region of *gbpB* [16]. A consensus binding sequence of CovR [30] was identified in promoters of *gbpB*, *wapE*, *lysM* and *epsC* (Fig. 2), further supporting direct regulation.

### Inactivation of VicRK- and/or CovR-regulated Genes does not Significantly Affect *S. mutans* Morphology or Cell Division during Planktonic Growth

To investigate whether the five newly identified genes of the VicRK and/or CovR regulons (*lysM*, *SMU.2146c*, *wapE, smaA* and *epsC*) function in cell wall biogenesis, knockout mutants of each gene were compared with the UA159 parent strain with regard to planktonic and biofilm growth, and cell and colony morphologies on MSA and BHI agar. Growth curves of all mutants in BHI were very similar to that of UA159 (data not shown). Additionally, SEM analysis of most mutants at mid-log (A_550 nm_ 0.3) and stationary (A_550 nm_ 0.9) growth phases did not reveal significant morphological changes (data not shown). The mutant in *epsC* (2.6-fold up-regulated in UAcov and not regulated by VicRK) showed smooth colony morphology on BHI and MSA agar (data not shown), while UAcov colonies were extremely rough and adhered tightly to the agar surface. Colony morphologies of UAvic and the other four mutants did not significantly differ from UA159 (data not shown). Because several mutants showed altered morphology during biofilm growth in the presence of sucrose (below), we examined whether sucrose would also affect morphogenesis of planktonic cells. However, addition of sucrose to culture media did not change the morphology of the mutants in the planktonic phase (data not shown).

### Inactivation of VicRK- and/or CovR-regulated Genes Affects *S. mutans* Biofilm Growth in the Presence of Sucrose

**Figure 3:**
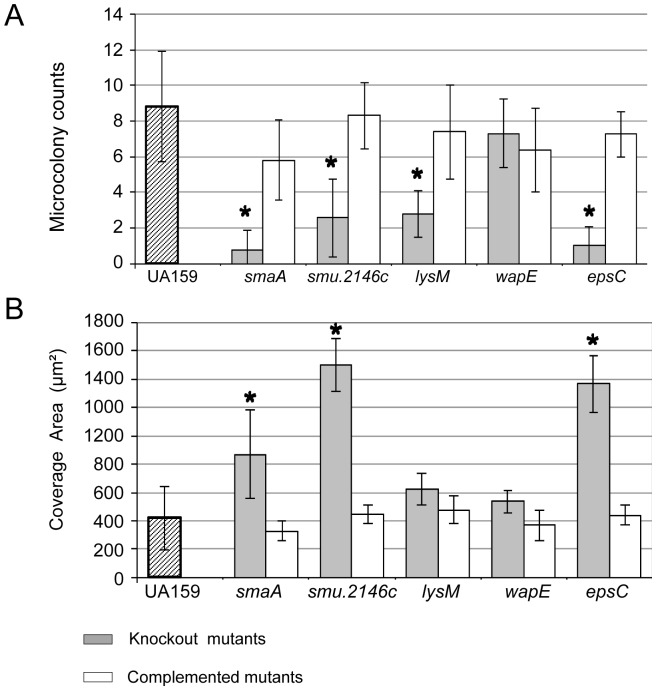
Quantitative comparisons of SEM images of 2 h biofilms using Image J software. (A) Columns represent mean numbers of matrix-based microcolonies, and (B) mean coverage areas (µm^2^) determined in 32 pre-determined areas per strain in one representative experiment. Bars indicate standard deviations. Asterisks indicate statistically significant differenced compared to parent UA159 (* p<0.05; Kruskal Wallis with *post hoc* Dunn's multiple comparison).

In contrast to the observations with planktonic cells, knockout mutants *smaA*, *SMU.2146c*, *lysM*, and *epsC* clearly showed different morphologic phenotypes compared to the UA159 parent strain, when grown as biofilms on glass slides for 2 to 4 h in the presence of sucrose (Fig. S2). After 2 h of biofilm formation, mutants UAsmaA, UA2146c and UAlysM formed chains significantly longer than those of UA159, a trait most prominent in the *lysM* mutant. In a total of 300 chains analyzed per strain, mean numbers of cocci per chain were 11.3 (±8.1), 10.0 (±7.8), 24.8 (±13.3) in UAsmaA, UA2146c and UAlysM, respectively, which were significantly higher compared to UA159 (mean: 6.0±4.3) (Kruskal-Wallis, p<0.05). Mutants UAsmaA, UA2146c, UAlysM and UAepsC were unable to form extracellular matrix-based microcolonies by 2 and 4 h, although some covered larger areas of the glass slides compared to UA159 (Fig. S2). To quantitatively analyze these phenotypes, mean numbers of microcolonies and mean covered areas of 2 h biofilms were determined using Image J software in 32 pre-determined areas per slide. As shown in Fig. 3A, UAsmaA, UA2146c, UAlysM, and UAepsC formed significantly lower numbers of microcolonies compared to parent, and *smaA*, *UA2146c* and *epsC* covered significantly larger areas (Fig. 3B). Most importantly, the biofilm phenotypes were completely restored in all complemented mutants (Fig. S2 and 3). Mutant *wapE* showed no clear alteration in biofilm phenotype, although tended to form longer chains (8.61±7.0) compared to UA159 (Fig. S2 and 4). The microcolony-defective phenotype of the mutants was not due to indirect effects on the genes responsible for the synthesis of and/or interaction with polysaccharides, i.e. *gtfB/C/D, gbpB/C* since their expression levels were not altered in the mutants compared to UA159 (data not shown).

**Figure 4:**
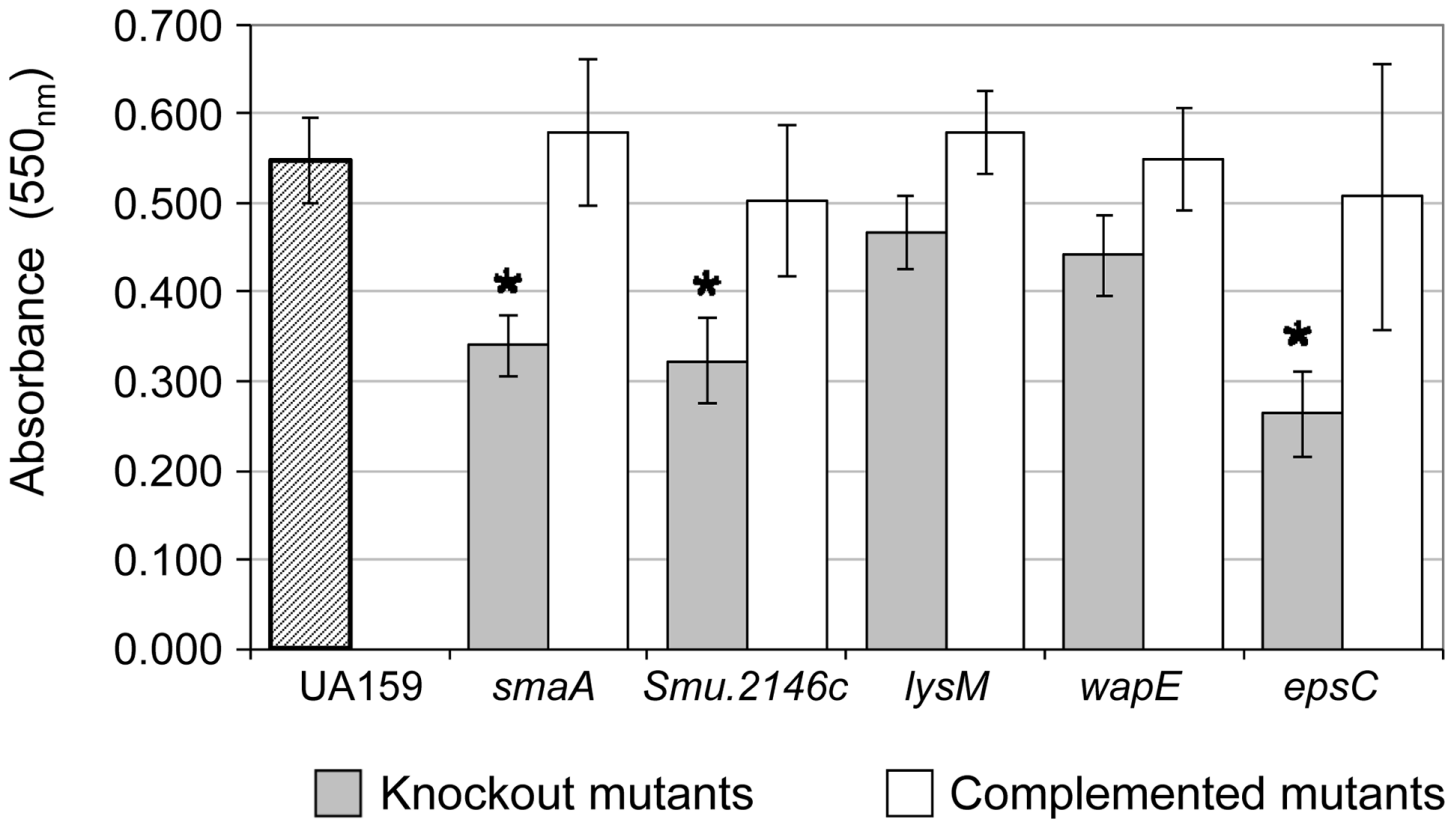
Comparison of biomasses of 8 h biofilms formed in the presence of sucrose. Columns represent means of three independent experiments performed in six replicates. Bars indicate standard deviations. Significant differences compared to parent strain UA159 (dashed column) are indicated by asterisks (* p<0.05; ANOVA with *post hoc* Dunnett‘s test).

Inactivation of VicR/CovR-regulated genes (*smaA*, *wapE, lysM, SMU.2146c, epsC)* did not significantly change the total biomass of 18 h biofilms in different media with 0.1% sucrose (data not shown), but changes in biofilm stability were noted in 8 h biofilms. Mutants in genes s*maA,SMU.2146c, epsC,* and, to a lesser extent *lysM* and *wapE*, formed biofilms that were loosely attached to microtiter wells, and detached during washing steps, differing from UA159 and complemented mutant biofilms (Fig. S4). Quantification of 8 h biofilms revealed significant reductions in biofilm biomass in mutants UAsmaA, UA2146c, and UAepsC, compared to UA159 (Fig. 4), while reductions in biofilm biomass of UAlysM and UAwapE did not reach statistical significance (Fig. 4).

UAvic shows impaired biofilm formation after 18 h in BHI with 0.1% sucrose [17]. Conversely, the UAcov mutant shows biofilms of higher biomass compared to the parent strain after 2, 4 (not shown) and 18 h of growth (means: 1.03±0.10 *versus* 0.50±0.06 respectively; ANOVA with *post hoc* Dunnett’s test: p<0.01). The enhanced biofilm biomass observed in UAcov differs from a previous report in which a *covR* mutant obtained in UA159 showed reduced biomass in 48 h biofilms formed in the presence of sucrose [13].

### Genes Involved in Cell Wall Biogenesis are Up-regulated during Biofilm Initiation and Sucrose Exposure

**Figure 5:**
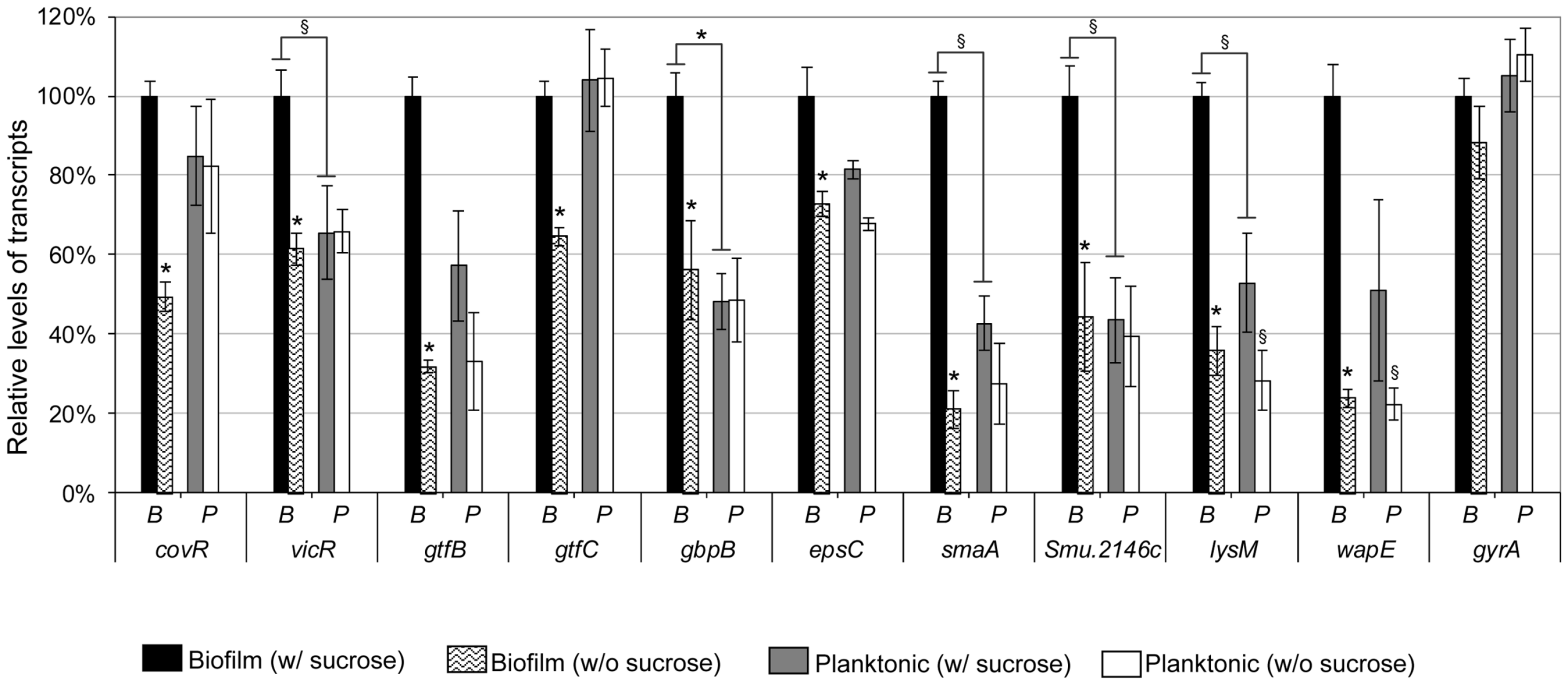
RT-qPCR analysis of gene expression in strain UA159 in 4 h biofilms and in planktonic phase. Biofilm (B) and planktonic (P) cells derived from the same cultures were grown in medium with or without 0.1% sucrose as indicated. Levels of transcripts in cells from sucrose-grown biofilms were set to 100% in order to calculate relative amounts of transcripts from cells grown under other conditions (biofilms w/o sucrose and planktonic cells). Columns represent means of three independent experiments performed in duplicate. Bars represent standard deviations. Statistically significant differences in gene expression in biofilm or planktonic cells grown without sucrose compared to the respective sucrose-grown cells are indicated above columns of cells without sucrose. Differences between biofilm and planktonic cells grown in the presence of sucrose are indicated above brackets. Statistical comparisons between biofilm *versus* planktonic cells in absence of sucrose are not shown. ANOVA with *post hoc* Dunnett’s test: * p<0.01; § p<0.05.

Given that the knockout mutants presented altered phenotypes associated with the initial phases of sucrose-dependent biofilm formation, we measured expression of their respective wild-type genes in the UA159 parent strain after 4 h of biofilm and planktonic growth with and without sucrose. As shown in Fig. 5, expression of *covR* and *vicR* increased approximately 1.6-2.0-fold in UA159 cells from 4 h sucrose-grown biofilms compared to those without this carbon source (ANOVA, Dunnett’s: p<0.01). In addition, *gtfB/C, gbpB* and the four novel VicR-regulated genes were significantly up-regulated in biofilms formed with sucrose compared to other conditions of growth. Means of 4.8 (79%), 2.2 (56%), 2.8 (64%) and 4.2-fold (76%) increases in expression of *smaA*, *SMU.2146c*, *lysM* and *wapE,* respectively, were observed in 4 h sucrose-grown biofilms, compared to biofilms grown without sucrose (ANOVA, Dunnett’s test: p<0.01) (Fig. 5). Up-regulation of the CovR-target *epsC* was detected in sucrose-grown biofilms to a smaller but still significant extent, compared to other VicR targets, which might be due to increased *covR* expression (Fig. 5). The g*bpB* gene was also up-regulated in UA159 biofilms in the presence of sucrose. Expression of the VicR targets was similar between planktonic cells grown with or without sucrose, except for *lysM* and *wapE*, which were significantly more expressed in the presence of sucrose (Fig. 5). Expression of *vicR*, *gpbB*, *smaA*, *Smu2146c* and *lysM* in biofilms formed in the presence of sucrose was significantly higher compared to sucrose-grown planktonic cells (Fig. 5) indicating that these genes are influenced by the mode of growth as well as by presence of sucrose. However, no significant changes in transcription of most genes was observed between biofilm and planktonic cells grown in the absence of sucrose, except for *gtfC* and *covR*, which were approximately 1.6 fold down- regulated (p<0.05) in biofilms compared to planktonic cells (Fig. 5). As a control, relative expression of *gyrA* was analyzed which did not show significant changes in all the conditions tested.

### VicRK and/or CovR-regulated Genes Involved in Cell Biogenesis Differentially Affect Mureinolytic and Cell Surface Properties, and Stress Sensitivity

**Figure 6:**
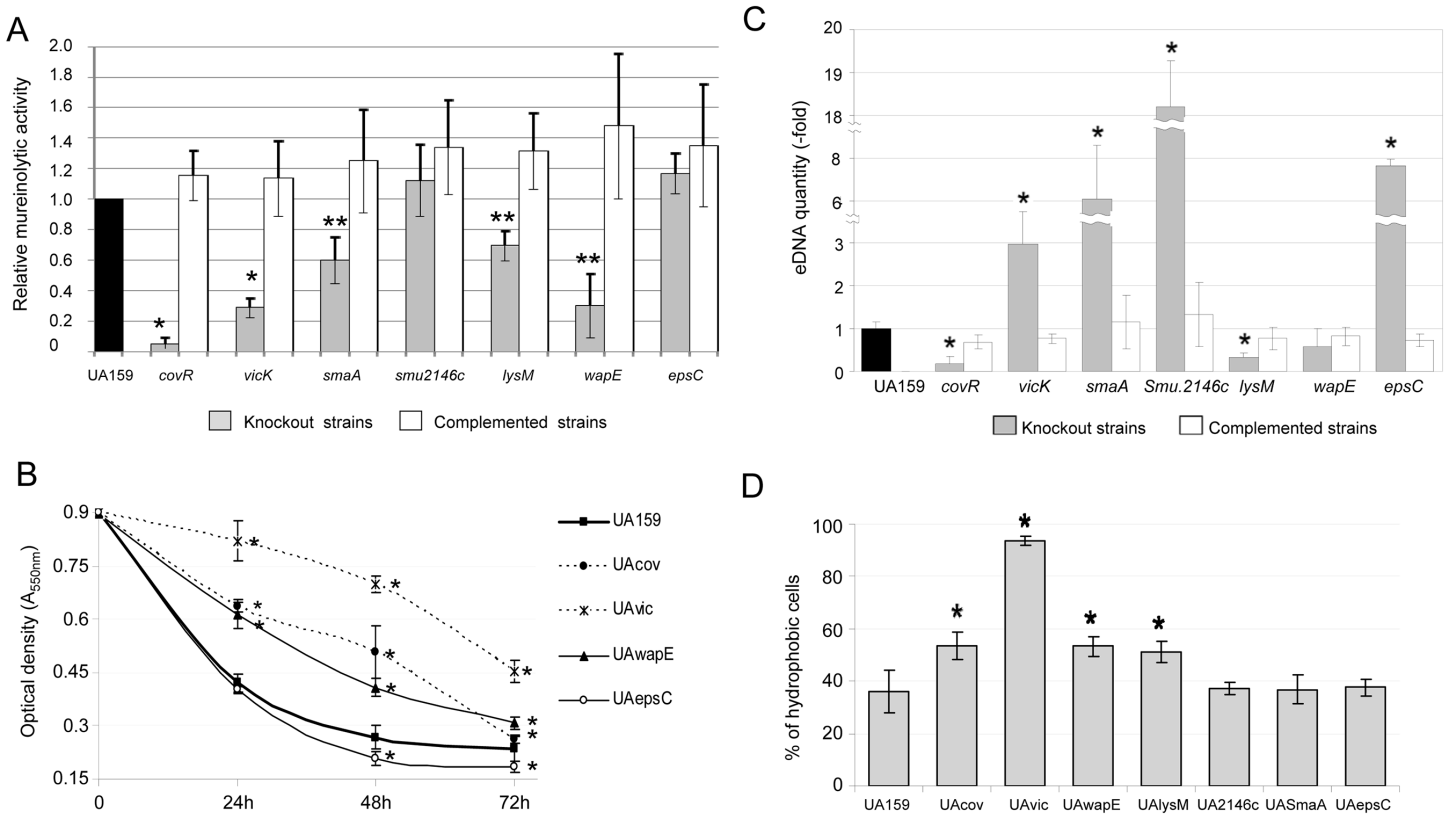
Comparisons of cell surface properties of UA159 and mutant strains. Columns represent means of: (A) mureinolytic activity, (B) autolysis, (C) amounts of extracellular DNA in culture supernatants of 8 h biofilms, and (D) cell surface hydrophobicities. Data were obtained from three independent experiments performed in triplicate. Bars represent standard deviations. Asterisks indicate significant differences (* p<0.01; **p<0.05) compared to control strains (parent or complemented), as tested by ANOVA with *post hoc* Dunnett. In B, only strains with significant differences compared to parent are shown.

The five novel proteins analyzed in this study have domains suggestive of functions linked to murein biogenesis or cell surface structure, which may also influence bacterial susceptibility to environmental stresses. Therefore, mutants in each gene were compared with UA159 and its *vicK*/*covR* derivatives with regard to lytic activity, cell surface properties and sensitivity to stress conditions. Inactivation of *vicK* and *covR* significantly reduced the mureinolytic activity of UA159, a phenotype completely restored in the respective complemented mutants (Fig. 6A). *WapE*, *lysM* and *smaA* mutants also showed impaired mureinolytic activity, implying a role of these genes in cell wall biogenesis. However, mutants UA2146c and UAepsC did not show significantly altered activities compared to the parent strain. Autolysis was significantly reduced only in UAvic, UAcov, and UAwapE (Fig. 6B). In addition, *epsC* mutant (up-regulated in UAcov) showed a small but significant increase in autolysis at 48 and 72 h compared to UA159 (Fig. 6B). Given that lytic activities on cell walls may promote the release of DNA to the extracellular environment, we measured amounts of eDNA in the culture medium of 8 h biofilms. Mutants with impaired lytic activities, UAcov, UAwapE, and UAlysM (but not UAvic and UAsmaA) showed significantly lower amounts of eDNA compared to parent (Fig. 6C). Curiously, culture supernatants of UA2146c biofilms showed extremely high amounts of eDNA compared to UA159, and significant increases in eDNA was also observed in UAepsC (Fig. 6C). Thus, inactivation of all identified genes significantly affected mureinolytic activities and/or release of DNA to culture medium.

WapE and LysM significantly influence cell hydrophobicity, and thus might contribute to the UAvic phenotype (Fig. 6D). An increased hydrophobicity was also observed in the *covR* mutant, while a mutant in the CovR-target *epsC* did not show changes in surface hydrophobicity (Fig. 6D). None of the novel VicR targets were involved in sensitivity to osmotic stress, except for *lysM*, since UAlysM was clearly resistant to osmotic stress (Fig. S4). Because *lysM* is down-regulated in UAvic, other yet unknown genes are likely involved in the sensitivity of this strain to osmotic stress. As previously observed [14], *vicK* inactivation did not significantly affect UA159 sensitivity to H_2_O_2._ However, *wapE*, a VicR-target, was significantly involved in resistance to oxidative stress (Kruskal-Wallis, p<0.01; Fig. S4). In summary, some of the new VicR/CovR target genes are involved in cell surface properties and sensitivity to osmotic and oxidative stress.

## Discussion

The roles of the TCS VicRK and CovRS have been investigated in several species of *Firmicutes*, including the major streptococcal pathogens *S. pneumoniae, S. pyogenes*, and *S. agalactiae* [31]–[34]. Both TCS regulate biogenesis of the cell surface in a species-specific manner [9], [32], [35]–[37], likely as result of evolutionary adaptation to the respective host/environmental niches. Changes in transcriptomic profiles of *vicK* and *covR* mutants are significantly influenced by nutritional and growth conditions [37]–[39], which may explain why some genes affected in this study of UAvic, e.g. *wapE,* and UAcov, e.g. SMU.2146c, were not detected in previous transcriptomic analysis of similar mutants of *S. mutans* [37]–[40]. *SMU.2146c/2147c* and *SMU.367* were detected in microarray analysis of a *vicK* mutant obtained in UA159 grown at pH 5.5, but the biological function of these genes was not investigated [38]. In *Bacillus subtilis* and in *S. pneumoniae*, VicK (YycG) is localized in the divisome and coordinates synthesis of autolysins and autolysin inhibitors during growth via kinase activation of VicR (YycF) [10]. In *S. mutans*, inactivation of *vicK* affects septum division, cell wall properties, biofilm formation and bacteriocin production [14], [16], [39] warranting an in-depth examination of gene functions associated with these phenotypes.

In the present study, we provide data that supports a model in which VicRK and CovR regulate several genes involved in cell wall biogenesis to optimize bacterial surface interactions with the extracellular matrix during sucrose-dependent biofilm growth in *S. mutans*. Defective biofilm phenotypes at 2, 4 and 8 h of growth were detected in mutants s*maA*, *SMU.2146c. lysM, and epsC* included impaired formation of microcolonies and/or biofilm fragility (Fig. 4, S2, S3), implying a significant influence of each gene in biofilm formation. An exception was *wapE*, whose inactivation promoted only a minor change in biofilm phenotypes (Fig. 4, S3), as described previously [41]. This observation may result from the static conditions under which biofilms were grown *in vitro* with high levels of glucan favoring increased biomass. Similarly, defects in microcolony formation or fragility detected in 2 to 4 h biofilms of UAlysM (Fig. S3) did not reflect significant changes in total biomass of 8 h biofilms (Fig. 4). Thus, analyses of mutants in multiple genes are needed to determine the contribution of *lysM* and *wapE* to biofilm formation.

Defining the specific functions of genes involved in cell wall biogenesis is important in order to clarify the transition from planktonic to biofilm growth in *S. mutans* in the presence of sucrose. Among the VicR-regulated genes (*smaA, lysM, wapE, SMU.2146c*), *smaA* showed mureinolytic activity, consistent with previous reports [31], as well as *lysM* and *wapE* (Fig. 6). LysM encodes a protein with a LysM domain, which is common in autolysins and likely involved in presentation of murein target sites for cleavage, e.g. cell wall septum [42]. Although no known mureinolytic domain is present in WapE, the protein has a N-terminal YSIRK lipoprotein signal motif which targets proteins to the septum in dividing Gram-positive bacteria [43] and thus WapE may coordinate cell division with cell surface biogenesis. *SMU.2146c* encodes a protein with an SLT transglycosylase domain known to degrade murein via cleavage of the β-1,4-glycosidic bonds of murein. Mutation in this gene did not impair mureinolytic activity or autolysis, but promoted release of large amounts of DNA during biofilm growth (Fig. 6), suggesting cell wall defects which may also be related to the long chain phenotype of UA2146c in biofilms (Fig. S2). Although DNA release reflects bacterial lysis [44], it can also occur in the absence of detectable autolysis in streptococcal species found in dental biofilms [45]. Interestingly, although changes in morphogenesis were evident in initial biofilms of mutants UAsmaA, UA2146c, UAlysM and UAepsC, (Fig. S2), these changes were weak or absent during planktonic growth in all mutants tested, indicating that there are cell biogenesis pathways specific to biofilm growth in *S. mutans.*

The associations between defects in biofilm initiation and fragility of 8 h biofilms were most noticeable in the presence of sucrose (Fig. 4), indicating defective interactions of cells with glucan or abnormal synthesis of glucan on the cell surface. Thus, the biofilm defective phenotype of the *vicK* mutant may result from accumulated changes in several protein functions, in addition to abnormal extracellular synthesis of polysaccharides by GtfB/C and Ftf [14] and cell-matrix interactions mediated by GbpB [16]. The up-regulation of *gtfB/C* during biofilm growth and in response to sucrose have been reported in several studies [46], [47]. In this study, we show that *gbpB, smaA*, *lysM, SMU.2146c* and *wapE* are also significantly up-regulated during the initial steps of biofilm formation (Fig. 5). Transcriptome studies of *S. mutans* biofilms formed on different surfaces, also detected up-regulation of *SmaA* and *SMU.574c* (adjacent to murein hydrolase exporter *SMU.575c* identified in this study; Table S2) [40] and *wapE* [41]. It is noteworthy that *wapE* and *lysM* are up-regulated even in planktonic cell populations exposed to sucrose compared to cells grown without sucrose (Fig. 5).

Sucrose is the only substrate for glucan synthesis by GtfB/C, and, to our knowledge, this is the first study showing that in *S. mutans* cell wall biogenesis genes coordinate sucrose-dependent biofilm formation under the direct control of VicRK and CovR. The VicK sensor kinase may auto-phosphorylate in response to as yet unknown environmental signals generated during biofilm growth and/or bacterial interaction with newly synthesized polysaccharides. Although amounts of VicR were not quantified, in UA159, *vicR* is up-regulated during initial biofilm growth in the presence of sucrose (Fig. 5), a finding also reported for strain GS5 [48]. Although *vicR* and *vicX* were down-regulated in UAvic, *vicR* self-regulation was not investigated in this study, and was not reported in other Gram-positive bacteria [49], [50]. We assume that in wild type cells VicR is activated by phosphotransfer from VicK, however, other TCS may also play a role in *vicR* expression, e.g. LiaFSR and ComCDE TCS [51], [52]. Data from this study suggest that growth of *S. mutans* in biofilms involves dynamic cooperation between VicR and CovR, since CovR directly represses several genes of the VicRK regulon, as well as *gbpC* and *epsC,* all involved in cell surface biogenesis and biofilm structuring (Table S2, Fig. 1/2; [18], [37]). Despite the direct interaction of r-CovR with the *gbpB* promoter (Fig. 1), increase in *gbpB* transcription in UAcov was relatively low (1.4-fold; p<0.05), compared to other CovR targets, e.g. *gpbC* (4.6-fold up-regulated) (Table S2). However, a greater increase in GbpB protein (4-fold) was found in UAcov compared to its transcript (Fig. S1), which might be explained by differences in transcript stabilities of CovR regulated genes. For example, *gbpC* up-regulation was approximately 2.2-fold in the *covR* mutant IBS132 [23] and 4.6-fold in UAcov (Table S2) although the half-life of these transcripts is very short (less than 2-8 min) in UA159 ([23], Fig. S1). Transcription profiles of UA159 *covR* mutants revealed that CovR acts both as a negative and a positive regulator ([37], Tables S2 and S3). Genes *wapE* and *gtfD* were significantly up-regulated in *covR* mutants obtained in strains UA159 (IBS10) and UA130 (GMS900), although direct binding of CovR to the respective promoters was not investigated [18]. We show that CovR directly represses the VicR-targets (*wapE* and *lysM*), but not *gtfD* (Table S2, Fig. 1 and 2). *EspC* was also directly repressed by CovR (Table S2; Fig. 1, 2), and since it is up-regulated during biofilm growth in the presence of sucrose (Fig. 5), this gene may also be controlled by other transcriptional factors. In Gram-positive bacteria, *epsC* encodes a UDP-N-acetylglucosamine (GlcNAc) 2-epimerase which catalyzes the reversible interconversion of UDP-GlcNAc and UDP-N-acetylmannosamine (UDP-ManNAc). The latter is required for attachment of teichoic acids to the cell wall, and for the biosynthesis of cell surface polysaccharides [53]. The lytic activity of the *espC* mutant was similar to that of UA159, but there was a significant increase in DNA release (Fig. 6), possibly due to increased cell wall permeability during biofilm growth. The UAepsC mutant showed significant defects in biofilm formation at 2, 4 and 8 h of growth (Fig. 3, 4, S2, S3), indicating a role of EpsC in sucrose-dependent biofilm formation.

VicR and CovR are members of the OmpR family, and as such contain a single winged helix structure for DNA binding [49], [54]. However, while VicR binds to a conserved DNA motif [49], which was found in all genes identified in this study (Fig. 2), a consensus target sequence for CovR was not discovered. In GAS, CovR binding depends on the structure assumed by CovR dimers formed after phosphorylation [54], [55] and the affinity of CovR-promoter interactions frequently involves phosphorylation-induced oligomerization to cover AT-rich DNA sequences [30], [54]. However, phosphorylation or cooperativity does not always affect CovR binding [54], [55]. A search for AT-rich consensus sequences in the promoters of the CovR-target genes identified in this study revealed several putative binding motifs (Fig. 2). In addition, EMSA assays show that VicR and CovR are able to co-bind the promoter regions of several genes (Fig. 1C).

In summary, the present study identified and characterized a set of new CovR and VicR targets involved in *S. mutans* cell wall/surface biogenesis, which are implicated in sucrose-dependent biofilm growth and structure. Further, we present evidence that CovR and VicR may interact cooperatively to coordinate functions of several genes, opening a new line of investigation to decipher roles for these important systems in the physiological transition from planktonic to biofilm growth of *S*. *mutans*.

## Supporting Information

Figure S1 **Effects of** ***covR*** **inactivation on GbpB production, and** ***gbpB*** **transcript stability.** (A) Relative amounts of GbpB in cell extracts (0.5 µg) and culture supernatants (2.5 µg) of mid-log phase culltures (A_550 nm_ 0.3) were determined by densitometry of western blots of GbpB probed with anti-GbpB antibody. Columns represent mean amounts of GbpB produced by *covR* mutant (UAcov) and its complemented mutant (UAcov+) compared to UA159 (set to 1). Results were calculated from three independent experiments. Bars represent standard deviations. Asterisks indicate significant differences compared to UA159 (* p<0.01) tested by ANOVA with *post hoc* Dunnett’s test. (B and C) Stability of *gbpB* and *gbpC* transcripts in UA159. Transcript amounts were measured in cultures of UA159 (A_550 nm_ 0.3) at 0, 2.5, 5, 10, 20, 30 and 60 min after addition of rifampin to block synthesis of RNA. (B) Representative northern blot (4 µg of RNA applied per lane). (C) Decay curve calculated using densitometric measures of northern blots from three independent experiments. Mean *gbpC* half-life (7.63 min ±1.36) was significantly lower compared to *gbpB* (20.33 min ±1.47) (T test, p<0.01).
(TIF)

Figure S2 **Biofilm phenotypes after 2 and 4 h of growth in the presence of sucrose.** Mutants of VicR and/or CovR-regulated genes are indicated below the respective panels. Complemented mutants are indicated by “+”. Biofilms of parent strain (UA159) are shown in the two panels at bottom.
(TIF)

Figure S3 **Biofilms formed during 8 h in the presence of sucrose.** Mutants of VicR/CovR-regulated genes (indicated above the respective panels) showed abnormal structure and detachment during washing steps of biofilms (arrows). These properties were not observed in parent UA159 or complemented mutants (indicated by “+”).
(TIF)

Figure S4 **Comparisons of sensitivities to stress conditions.** Decreases in cell viability of UA159 and mutants in VicR and/or CovR target genes were measured after osmotic (A) and oxidative (B) stresses. Columns represent means obtained from three independent experiments performed in triplicate. Asterisks indicate significant differences compared to UA159 (* p<0.01) tested by ANOVA with *post hoc* Dunnett’s test (A) or by Kruskal-Wallis (B).
(TIF)

Table S1 **Oligonucleotides used in this study.**
(DOC)

Table S2 **Comparative transcriptional profiles of** ***vicK*** **(UAvic) and** ***covR*** **(UAcov) mutants with parent strain UA159.**
(DOC)

Table S3 **Additional genes significantly down-regulated in UAcov. (cut-off 2.5 fold).**
(DOC)
