# Supplementary figures and images for: CovR and VicRK Regulate Cell Surface Biogenesis Genes Required for Biofilm Formation in Streptococcus mutans
Source: PLoS One. 2013 Mar 12;8(3):e58271. doi: 10.1371/journal.pone.0058271 (PMC3595261; doi:10.1371/journal.pone.0058271)

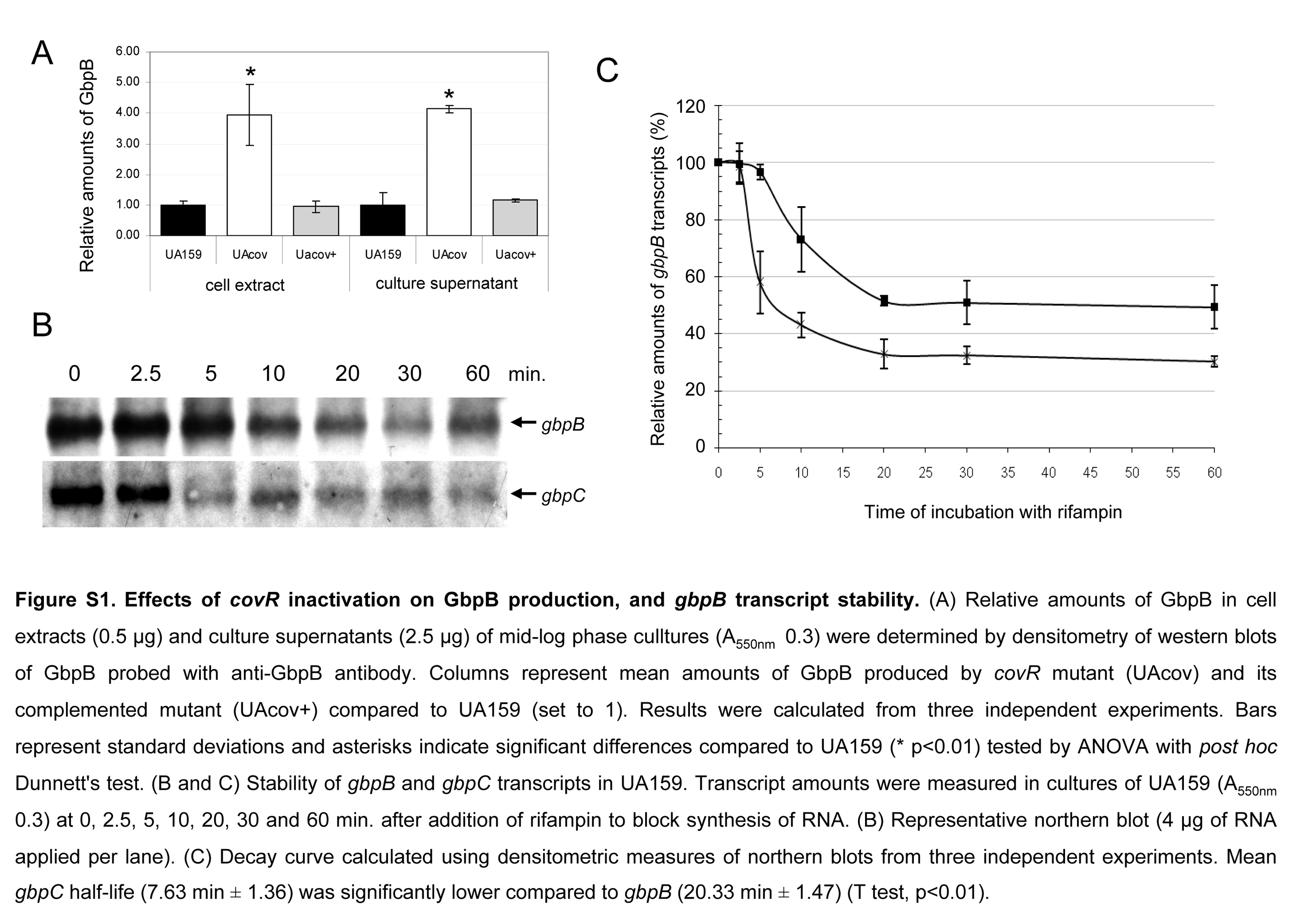

Supplement: Figure S1 — Effects of covR inactivation on GbpB production, and gbpB transcript stability. (A) Relative amounts of GbpB in cell extracts (0.5 µg) and culture supernatants (2.5 µg) of mid-log phase culltures (A550 nm 0.3) were determined by densitometry of western blots of GbpB probed with anti-GbpB antibody. Columns represent mean amounts of GbpB produced by covR mutant (UAcov) and its complemented mutant (UAcov+) compared to UA159 (set to 1). Results were calculated from three independent experiments. Bars represent standard deviations. Asterisks indicate significant differences compared to UA159 (* p<0.01) tested by ANOVA with post hoc Dunnett’s test. (B and C) Stability of gbpB and gbpC transcripts in UA159. Transcript amounts were measured in cultures of UA159 (A550 nm 0.3) at 0, 2.5, 5, 10, 20, 30 and 60 min after addition of rifampin to block synthesis of RNA. (B) Representative northern blot (4 µg of RNA applied per lane). (C) Decay curve calculated using densitometric measures of northern blots from three independent experiments. Mean gbpC half-life (7.63 min ±1.36) was significantly lower compared to gbpB (20.33 min ±1.47) (T test, p<0.01). (TIF) [file pone.0058271.s001.tif]

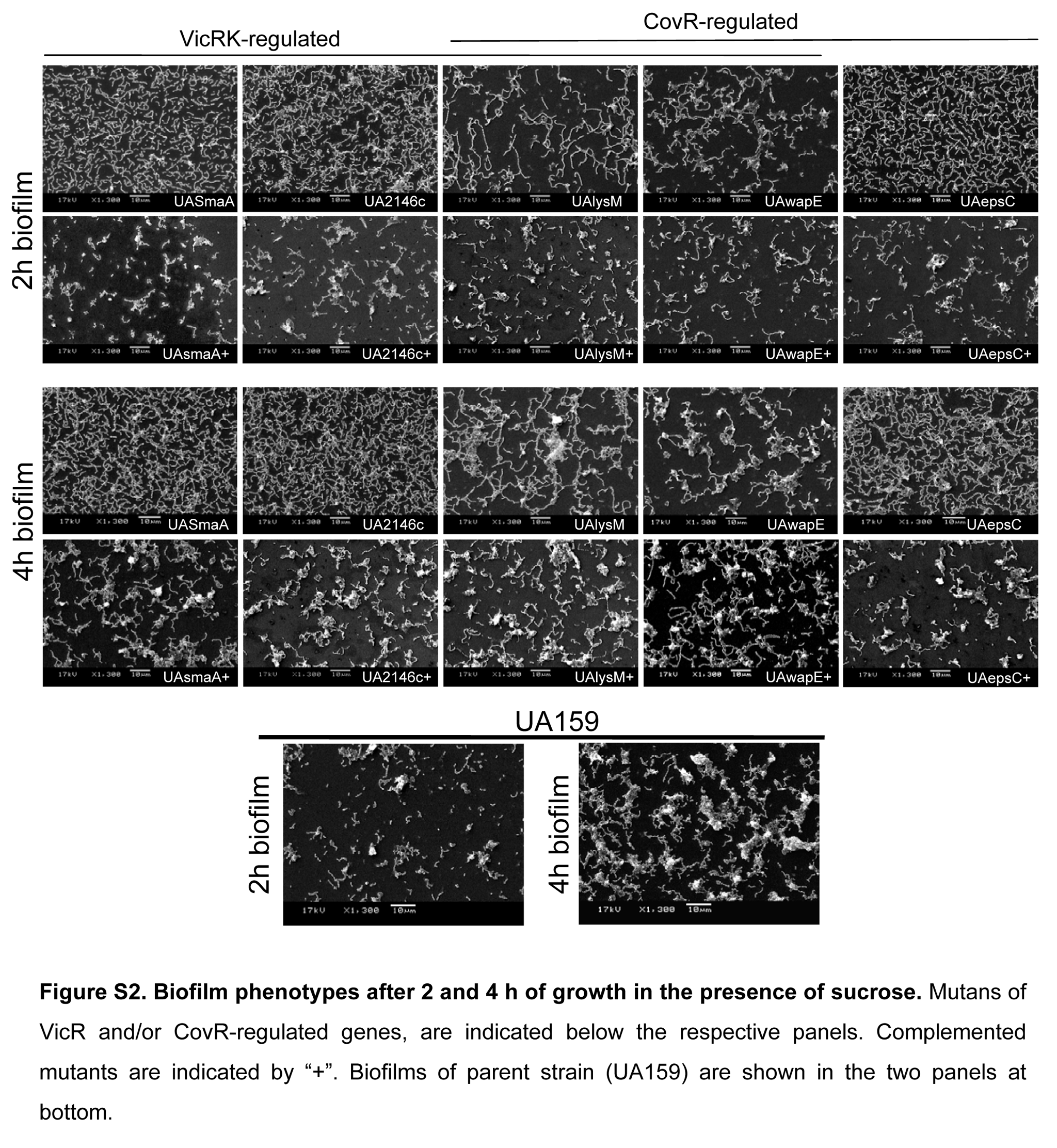

Supplement: Figure S2 — Biofilm phenotypes after 2 and 4 h of growth in the presence of sucrose. Mutants of VicR and/or CovR-regulated genes are indicated below the respective panels. Complemented mutants are indicated by “+”. Biofilms of parent strain (UA159) are shown in the two panels at bottom. (TIF) [file pone.0058271.s002.tif]

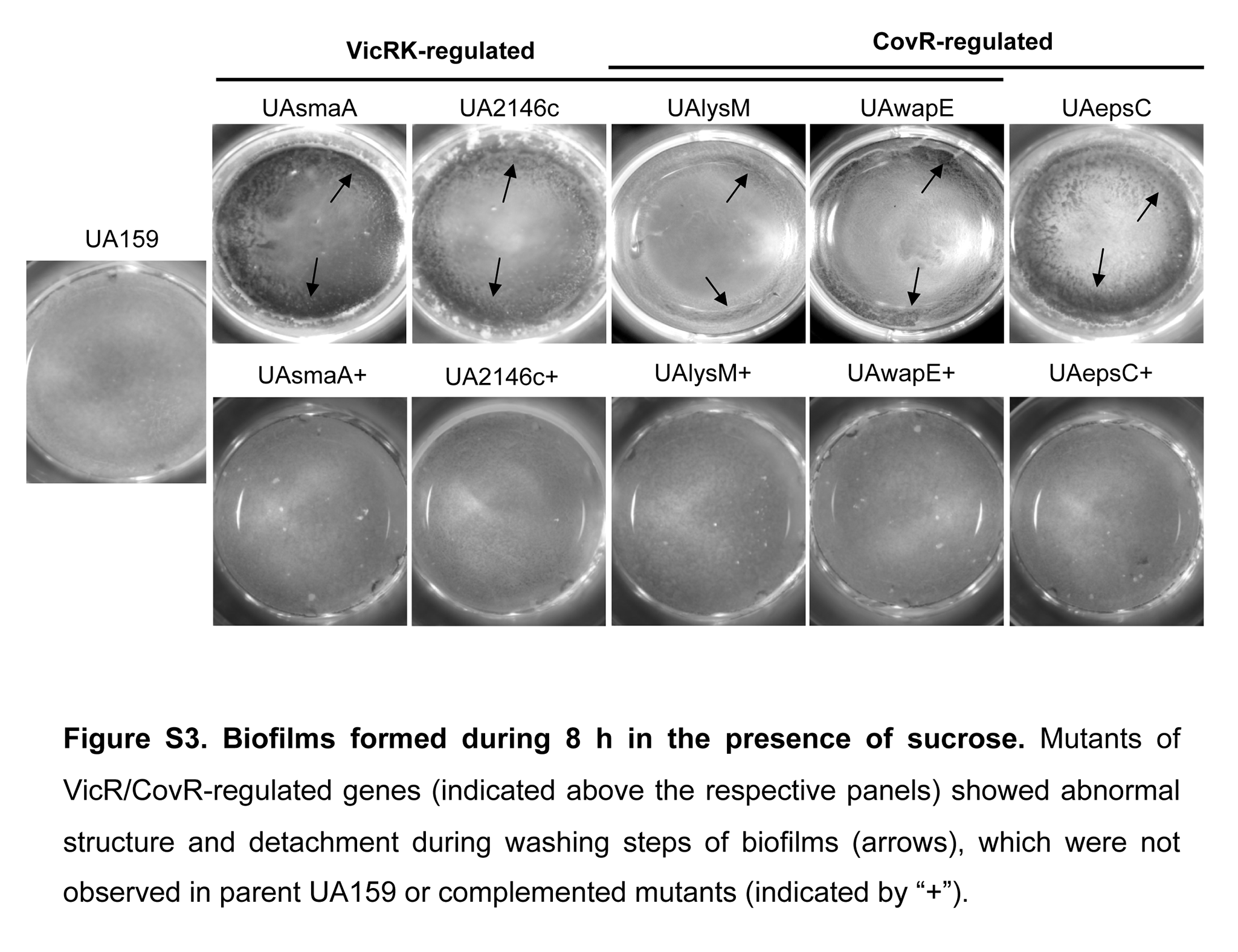

Supplement: Figure S3 — Biofilms formed during 8 h in the presence of sucrose. Mutants of VicR/CovR-regulated genes (indicated above the respective panels) showed abnormal structure and detachment during washing steps of biofilms (arrows). These properties were not observed in parent UA159 or complemented mutants (indicated by “+”). (TIF) [file pone.0058271.s003.tif]

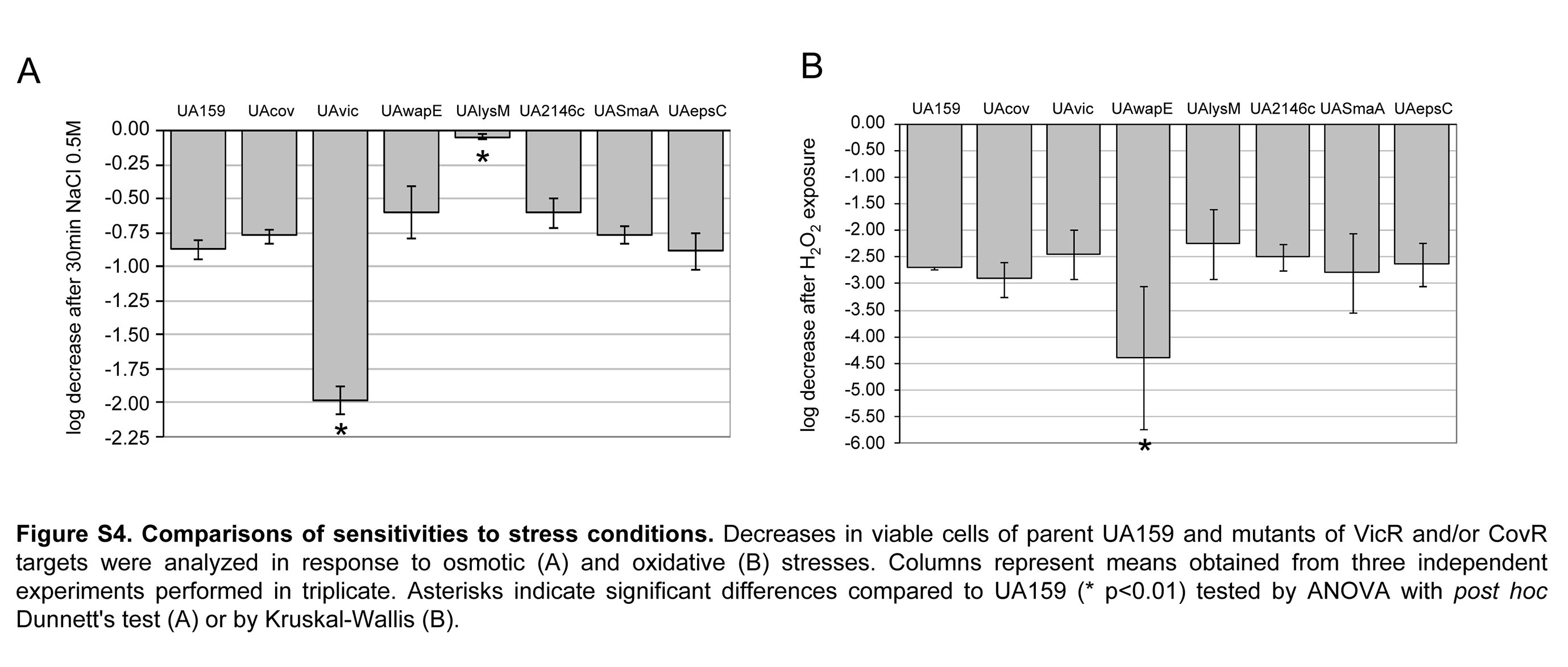

Supplement: Figure S4 — Comparisons of sensitivities to stress conditions. Decreases in cell viability of UA159 and mutants in VicR and/or CovR target genes were measured after osmotic (A) and oxidative (B) stresses. Columns represent means obtained from three independent experiments performed in triplicate. Asterisks indicate significant differences compared to UA159 (* p<0.01) tested by ANOVA with post hoc Dunnett’s test (A) or by Kruskal-Wallis (B). (TIF) [file pone.0058271.s004.tif]
