# Supplementary material for: CovR and VicRK Regulate Cell Surface Biogenesis Genes Required for Biofilm Formation in Streptococcus mutans
Source: PLoS One. 2013 Mar 12;8(3):e58271. doi: 10.1371/journal.pone.0058271 (PMC3595261; doi:10.1371/journal.pone.0058271)
Supplement: Table S1 — Oligonucleotides used in this study. (DOC) [file pone.0058271.s005.doc]

**Table S1. Oligonucleotides used in this study.**

| Primer names | Sequence 5’-3’^a^  (Forward/Reverse) | Product size, gene (upstream/internal gene position), comment or reference. | | |
| --- | --- | --- | --- | --- |
| **Mutant/plasmid constructs** | | | | |
| E1-*AscI* / E2-*XhoI* | TTGGCGCGCCTGGCGGAAACGTAAAAGAAG / TTCTCGAGGGCTCCTTGGAAGCTGTCAGT | 998 bp, amplicon containing the *erm^r^* gene from pVA838 | | |
| wapP1 / wapP2-*AscI* | TGGTATCTACAAACAACAGGACA / TTGGCGCGCCGTCGTTAAAATAGCTCCACAAAG | 541 bp, *SMU.1091* | | |
| wapP3-*XhoI* / wapP4 | TTCTCGAGTTCTCTTTCTGCTCAACTACCTG / ACTAGAGTCAAGAGAGCAGTTCG | 535 bp, *SMU.1091* | | |
| lysP1 / lysP2-*AscI* | TCAGTTTAGCAGATGCAGTCG / TTGGCGCGCCCTAGCTGCTGTTGCAATTCCT | 673 bp, *SMU.2147c* | | |
| lysP3-*XhoI* / lysP4 | TTCTCGAGGAGTGATTCTTACTTGAATGGTG / ACTTCTTGAGCTTGTGCAGTGT | 472 bp, *SMU.2147c* | | |
| 2146P1 / 2146P2-*AscI* | GAGTGATTCTTACTTGAATGGTGA / TTGGCGCGCCAAAGGTAAAACCCAAAATTGAC | 369 bp, *SMU.2146c* | | |
| 2146P3-*XhoI* / 2146P4 | TTCTCGAGAATGCTTATAAAGCCCAAGGT / AGCCACATCTTTGTAATCTTCTG | 598 bp, *SMU.2146c* | | |
| smaP1 / maP2-*AscI* | AATGAATACAATGCGGAGGTT / TTGGCGCGCCACACAGCCAATTAAAACAGATG | 565 bp, *SMU.609* | | |
| smaP3-*XhoI* / smaP4 | TTCTCGAGGCAGGATGACATTGTCTGGTA / TTAGGGGCTTCTACTGTCGTT | 500 bp, *SMU.609* | | |
| epsP1 / epsP2-*AscI* | TGATAAAGAAACTTCGGTAATGG / TTGGCGCGCCTTTCAAAACGTTCAGTCTGCT | 533 bp, *SMU.1437c* | | |
| epsP3-*XhoI* / epsP4 | TTCTCGAGAGCAAATACAGAGCGTATCCA / AAGCTAAACGGTCATACAAAGG | 653 bp, *SMU.1437c* | | |
| wapC1-*EcoRI* / wapC2-*BamHI* | TTGAATTCTGGTATCTACAAACAACAGGACA / TTGGATCCACTAGAGTCAAGAGAGCAGTTCG | 2361 bp amplicon containing the *wapE* gene. Used for complementation. | | |
| lysC1-*SacI* / lysC2-*BamHI* | TTGAGCTCTCAGTTTAGCAGATGCAGTC / TTGGATCCACTTCTTGAGCTTGTGCAGTGT | 1803 bp amplicon containing the *lysM* gene. Used for complementation. | | |
| 2146C1-*EcoRI* / 2146C2-*BamHI* | TTGAATTCGAGTGATTCTTACTTGAATGGTGA / TTGGATCCAGCCACATCTTTGTAATCTTCTG | 1461 bp amplicon containing the *SMU.2146c* gene. Used for complementation. | | |
| smaC1-*EcoRI* / smaC2-*BamHI* | TTGAATTCAATGAATACAATGCGGAGGTT / TTGGATCCGCTGCTATTTTTCGAGCACA | 2413 bp amplicon containing the *smaA* gene. Used for complementation. | | |
| epsC1-*EcoRI* / epsC2-*BamHI* | TTGAATTCTGATAAAGAAACTTCGGTAATGG / TTGGATCCAAGCTAAACGGTCATACAAAGG | 1845 bp amplicon containing the *epsC* gene. Used for complementation. | | |
| covRHisF-*NcoI* / covRHisR-*XhoI* | AACCATGGCTAAGGACATTTTAATTATTGAA / TTCTCGAGATTATTTTCGCGAATGATATACCC | 702 bp, *covR* ORF, His-Tag fusion construction | | |
| vicRHisF-*NcoI* / vicRHisR-*XhoI* | AACCATGGAGAAAATTCTAATCGTTGACGA / AACTCGAGGTCATATGATTTCATGTAATAAC | 717 bp, *vicR* ORF, HisTag fusion construction | | |
| **qPCR analysis** | | | | |
| 16SRNA | CGGCAAGCTAATCTCTGAAA / GCCCCTAAAAGGTTACCTCA | 190 bp [19] | | |
| SMU.22 *(gbpB)* | CAACAGAAGCACAACCATCA / TGTCCACCATTACCCCAGT | 151 bp [19] | | |
| SMU.367 | CGAATGCCAATACTTATCCTGT / ATGTCCATAACCACCATCTGTC | 185 bp | | |
| SMU.391c | CATCTTCTTAGGCTGCGGTAT / ACAGTCTATTTTGATGGCACATT | 213 bp | | |
| SMU.575c | ATTTCCCTAGCAGCCAATTTA / AGCTATGAGCAGTCCATTTTTC | 175 bp | | |
| SMU.609 | GGCACAAGGAACCTATCACTTT / GCTTTCCAATAACAACATAACGAC | | 191 bp | |
| SMU.772 | TCAAAGCCATTTCACTTCTACAG / AGAGTCATACCGCCACCATTA | | 198 bp | |
| SMU.910 *(gtfD)* | TGATTCGTGGTATCGTCCTAA / GTTGAGACTTTCTTGGCTGCT | | 199 bp | |
| SMU.992 | GCAGTTCACTAAGACAGCATCC / CAAGCCAAGCAAACCTAAGAG | | | 230 bp |
| SMU.1004 *(gtfB)* | CGAAATCCCAAATTTCTAATGA / TGTTTCCCCAACAGTATAAGGA | | | 197 pb [19] |
| SMU.1005 (*gtfC*) | ACCAACCGCCACTGTTACT / AACGGTTTACCGCTTTTGAT | | | 161 bp [19] |
| SMU.1006 | ATTACCAATCCGCAAATCCTT / TGGTGAAATATCCGTCCATCT | | | 194 bp |
| SMU.1091 | TATTCCTGTGCCTTCTGTTGA / GCCTTCTTGACTTTTGGATTG | | | 187 bp |
| SMU.1117 | AAGGAAAGCACCATTACGGA / GAATTAGCATTCGGAGAAACTG | | | 174 bp |
| SMU.1334c | TTGGCTAATGGCACATACAAC / TCTCTTTTAGGGCACCTTTTATC | | | 174 bp |
| SMU.1342c | TGTATTTCACTCTCTCCTCTGG / ATGACTACAACCGAACTTATGC | | | 199 bp |
| SMU.1395c | ATGACAGATCAAGAATTAGAACA / ATATTCAGGAAATGCTTCATC | | | 114 bp |
| SMU.1396 (*gbpC*) | TGTAGCTGTTGTCGAAAGTCA / CCTGTGCTTTGTTATCTGCAT | | | 178 bp |
| SMU.1434c | TTGGTCTATTTAAGCCAGGTGT / AGTAAGCCAATAAGGCAATCC | | | 189 bp |
| SMU.1437c | GCAAATACAGAGCGTATCCATC / TAAAGTCCCTGCTGCTACTCC | | | 189 bp |
| SMU.1489 | TCTTGAAAGGGCAGAAATACC / AAGGACTCTTATTGGCTGTTGA | | | 183 bp |
| SMU.1516 | CGGCGTGATGAATATGATGAA / GAGGTTAATGGTGTCCGCAGT | | | 185 bp |
| SMU.1517 (*vicR*) | AGTGGCTGAGGAAAATGCTT / CATCACCTGACCTGTGTGTG | | | 163 bp |
| SMU.1599 | CTATGGTAGTGCTCAAATGCTG / CTGTCAAGAAAACCGAAACTG | | | 194 bp |
| SMU.1881c | GGTTCTGGTAAAACGACTCTTG / GCTTGTTGGGGGAGATAGTTA | | | 143 bp |
| SMU.1896c | CAGATACTGTCCCAGCCAAAA / TATGATGGAAATAAAGGCGTTG | | | 93 bp |
| SMU.1910c | AAGAGGACCAGTCGGTTAAAG / CGAACCAAGGACTATTAAAAACA | | | 173 bp |
| SMU.1918c | TTTGTAGCTGGCCTTAGTCAA / GCCAAGATAATAGCAGAAAAATG | | | 152 bp |
| SMU.1919c | CCGATGACTTTCACTTTTCTCT / ATAATCAGGACGATGTTGGACT | | | 191 bp |
| SMU.1988c | GTTATCAAAAAGAGTGGGCAAA / AGTTAAAAGCGCAAGAGAATGA | | | 155 bp |
| SMU.2028 | ATCACCCTGTTGTGTCATTTCT / GGCGACTTACTCTTATTATGCTGT | | | 190 bp |
| SMU.2146c | AATCTGTTCTTGCTCACACTGC / ACATTATCAGTTGGTTCAGTTGCT | | | 145 bp |
| SMU.2147c | TTATCAGAGATTGCTTCAACACA / CTGAGGTTTCTGCTTCATTTATC | | | 175 bp |
| **EMSA** | | | | |
| SMU.22 *(gbpB)* | TTGACAGCTTATCCTTTAAATG / TTTACAGCTGATAATGTTGTCG | | | 300 (223/87 bp) |
| SMU.367 | CGTGGTCCTAGTCTTGTTATTTG / CTGCATCAGCCATTTTATTTC | | | 328 (240/88 bp) |
| SMU.609 | TGGAAGAGAAGATGTAACAATGA / ACACAGCCAATTAAAACAGATG | | | 359 (270/89 bp ) |
| SMU.910 (*gtfD*) | TCTCTCCTGACCACTCCCTTA / TACCCAGTGCTTTTTAACCTTG | | | 324 (273/51 bp) |
| SMU.1005 | GATGCTAACTCTGGAGAACGA / TCCTGAAAGAGAGGTCAAAGTC | | | 330 (231/ 99 bp) |
| SMU.1091 | GCTTCCGAACATTGATCTTATT / GTCGTTAAAATAGCTCCACAAAG | | | 324 (256/68 bp) |
| SMU.1434c | GTCTTGGCATCAGTCTCTTTTT / GTGCAATCACAACATCTTCATT | | | 298 (249/49 bp) |
| SMU.1437c | TTGACATATACGAAACAGTCTCT / TTGTACCAAAGACAAGCATAA | | | 311 (274/37 bp) |
| SMU.1924 (*covR*) | AGATGTCCTCTACCCATTGAAAAATGG / AACCTCATATCCTTCATGTTGTAATTCTAAAG | | | 356 (269/87 bp) [12] |
| SMU.2147c | ACAGCTTGGCTATTGTTGGTA / TGCAATTCCTGCAAAACTAAC | | | 316 (250/66 bp) |

^a^ Underlined sequences indicate restriction enzyme linkers.
