# Supplementary material for: CovR and VicRK Regulate Cell Surface Biogenesis Genes Required for Biofilm Formation in Streptococcus mutans
Source: PLoS One. 2013 Mar 12;8(3):e58271. doi: 10.1371/journal.pone.0058271 (PMC3595261; doi:10.1371/journal.pone.0058271)
Supplement: Table S2 — Comparative transcriptional profiles of vicK (UAvic) and covR (UAcov) mutants with parent strain UA159. (DOC) [file pone.0058271.s006.doc]

**Table S2. Comparative transcriptional profiles of *vicK* (UAvic) and *covR* (UAcov) mutants with parent strain UA159.**

| **Gene ID (NCBI)** | **Encoded protein; conserved domains** | | **UAvic** | | | **UAcov** | |
| --- | --- | --- | --- | --- | --- | --- | --- |
|  |  | | **Microarray fold** | **qPCR fold** | | **Microarray fold** | **qPCR fold** |
| **Hypothetical proteins with no conserved domains** | | | | | | | |
| SMU.175 | No conserved domains identified | | -3.1 |  | |  |  |
| [SMU.391c](http://genome.brop.org/modules.php?op=modload&name=GenomeExp&file=index&option=ncbi&gsource=oral&org=smut&gprog=gview&geneid=SMU.391c) | No conserved domains identified | |  |  | | +2.5 | +2.5^†^ |
| SMU.992 | No conserved domains identified | | -6.1 | -7.9^†^ | |  |  |
| SMU.1395c | No conserved domains identified | |  |  | | +20.7 | +3.0^†^ |
| [SMU.1435c](http://genome.brop.org/modules.php?op=modload&name=GenomeExp&file=index&option=ncbi&gsource=oral&org=smut&gprog=gview&geneid=SMU.1435c) | No conserved domains identified | |  |  | | +4.8 |  |
| [SMU.1436c](http://genome.brop.org/modules.php?op=modload&name=GenomeExp&file=index&option=ncbi&gsource=oral&org=smut&gprog=gview&geneid=SMU.1436c) | No conserved domains identified | |  |  | | +4.8 |  |
| [SMU.1910c](http://genome.brop.org/modules.php?op=modload&name=GenomeExp&file=index&option=ncbi&gsource=oral&org=smut&gprog=gview&geneid=SMU.1910c) | No conserved domains identified | |  |  | | +2.6 | +1.9^‡^ |
| [SMU.1919](http://genome.brop.org/modules.php?op=modload&name=GenomeExp&file=index&option=ncbi&gsource=oral&org=smut&gprog=gview&geneid=SMU.1919)c | No conserved domains identified | |  |  | | +3.5 | +5.8^†^ |
| **Biogenesis of and/or interaction with extracellular matrix** | | | | | | | |
| SMU.22* | GbpB; peptidoglycan hydrolase activity domain | | -3.4 | -2.2^†^ | | NS | +1.4^‡^ |
| SMU.772 | GbpD; Glucan-binding and esterase/lipase domains | | -2.2 | -1.9^‡^ | |  |  |
| SMU.910 | GtfD; Glucan-binding and dextransucrase-like domain | | -6.1 | -14.1^†^ | |  |  |
| [SMU.1004](http://genome.brop.org/modules.php?op=modload&name=GenomeExp&file=index&option=ncbi&gsource=oral&org=smut&gprog=gview&geneid=SMU.1004)* | GtfB; Glucan-binding and dextransucrase-like domain | | -1.8 | -2.9^†^ | | +22.0 | +18.0^†^ |
| [SMU.1005](http://genome.brop.org/modules.php?op=modload&name=GenomeExp&file=index&option=ncbi&gsource=oral&org=smut&gprog=gview&geneid=SMU.1005)* | GtfC; Glucan-binding and dextransucrase-like domain | | NS | -1.4^‡^ | | +2.5 | +4.5^†^ |
| [SMU.1396](http://genome.brop.org/modules.php?op=modload&name=GenomeExp&file=index&option=ncbi&gsource=oral&org=smut&gprog=gview&geneid=SMU.1396) | GbpC; Glucan binding and LPXTG domains | |  |  | | +12.4 | +4.6^†^ |
| [SMU.1432c](http://genome.brop.org/modules.php?op=modload&name=GenomeExp&file=index&option=ncbi&gsource=oral&org=smut&gprog=gview&geneid=SMU.1432c) | endoglucanase; Glycosyl hydrolase family 8 domain | |  |  | | +5.2 |  |
| SMU.2028* | Ftf; Levansucrase activity domain | | -4.5 | -3.9^†^ | | +2.5 | +1.5^‡^ |
| **Cell wall or cell envelope biogenesis** | | | | | | | |
| └ ***Cell wall biogenesis*** | | | | | | | |
| SMU.367 | SceB; Lysin and CHAP amidase domains | | -2.2 | -1.5^‡^ | |  |  |
| [SMU.575c](http://genome.brop.org/modules.php?op=modload&name=GenomeExp&file=index&option=ncbi&gsource=oral&org=smut&gprog=gview&geneid=SMU.575c) | YsbA; LrgA murein hydrolase exporter domain | |  |  | | +7.2 | +4.5^†^ |
| SMU.609 | SmaA; SH3b murein hydrolase and BSP-like domains **^a^** | | +16.1 | +13.7^†^ | |  |  |
| SMU.1006 | SMU.1006; SalX ABC-type domain **^b^** | |  |  | | +5.9 | +3.0^†^ |
| SMU.1091* | WapE; YSIRK signal (cross-wall-targeting lipoprotein signal motif) domain | | -2.8 | -1.7^‡^ | | +2.6 | +1.8^‡^ |
| [SMU.1434c](http://genome.brop.org/modules.php?op=modload&name=GenomeExp&file=index&option=ncbi&gsource=oral&org=smut&gprog=gview&geneid=SMU.1434c) | WcaA; CESA-like (cellulose synthase superfamily) domain | |  |  | | +4.3 | +2.9^†^ |
| [SMU.1437c](http://genome.brop.org/modules.php?op=modload&name=GenomeExp&file=index&option=ncbi&gsource=oral&org=smut&gprog=gview&geneid=SMU.1437) | EpsC; wecB (UDP-N-Acetylglucosamine 2-Epimerase) domain | |  |  | | +3.5 | +2.6^†^ |
| [SMU.1918](http://genome.brop.org/modules.php?op=modload&name=GenomeExp&file=index&option=ncbi&gsource=oral&org=smut&gprog=gview&geneid=SMU.1918)c | DedA; SNARE-like domain **^c^** | |  |  | | +4.9 | +3.0^†^ |
| SMU.2146c | SMU.2146c; Transglycosylase SLT domain **^d^** | | -15.2 | -15.2^†^ | |  |  |
| SMU.2147c* | LysM; Lysin and NlpD domains | | -3.4 | -3.5^†^ | | +2.6 | +1.8^†^ |
| └ ***Lipid metabolism*** | | | | | | | |
| [SMU.438c](http://genome.brop.org/modules.php?op=modload&name=GenomeExp&file=index&option=ncbi&gsource=oral&org=smut&gprog=gview&geneid=SMU.438c) | [SMU.438c](http://genome.brop.org/modules.php?op=modload&name=GenomeExp&file=index&option=ncbi&gsource=oral&org=smut&gprog=gview&geneid=SMU.438c); HSP70-class ATPase domain |  | | |  | +3.1 |  |
| SMU.1334c* | Sfp; Spf (phosphopantetheinyl transferase) domain | -3.2 | | | -2.8^†^ | -3.6 |  |
| SMU.1335c* | enoyl-ACP reductase; enACPred_II domain | -3.1 | | | -3.4^†^ | -3.7 |  |
| SMU.1336c* | PksD; Acyl transferase domain | -3.5 | | | -4.6^†^ | -3.7 |  |
| **Transporters** | | | | | | | |
| SMU.1338c | SMU.1338c permease; H+ Antiporter-like domain | -3.6 | | |  | -4.2 |  |
| SMU.1982c | SMU.1982c; PulG-secretory-like domain | -3.4 | | | -1.9^‡^ |  |  |
| **Stress response** | | | | | | | |
| SMU.1117 | NaoX; NADH oxidase-like domain |  | | |  | +3.1 | +2.2^†^ |
| **DNA replication/recombination and competence** | | | | | | | |
| SMU.191c | SMU.191c integrase; PhiLC3-related integrase domain | -4.5 | | |  |  |  |
| SMU.198c | SMU.198c transposon; AAA-like domain **^e^** | -3.9 | | |  |  |  |
| [SMU.1353](http://genome.brop.org/modules.php?op=modload&name=GenomeExp&file=index&option=ncbi&gsource=oral&org=smut&gprog=gview&geneid=SMU.1353) | [SMU.1353](http://genome.brop.org/modules.php?op=modload&name=GenomeExp&file=index&option=ncbi&gsource=oral&org=smut&gprog=gview&geneid=SMU.1353) transposase; Integrase-like core domain |  | | |  | +2.7 |  |
| SMU.1895c | ComC; ComC-like domain | -25.3 | | |  |  |  |
| SMU.1921c | DnaI; DnaI_N domain |  | | |  | +4.2 |  |
| [SMU.1922](http://genome.brop.org/modules.php?op=modload&name=GenomeExp&file=index&option=ncbi&gsource=oral&org=smut&gprog=gview&geneid=SMU.1922)c | DnaB; DnaB_COG3611 domain |  | | |  | +4.6 |  |
| **Transcription factors and regulators** | |  | | |  |  |  |
| [SMU.112c](http://genome.brop.org/modules.php?op=modload&name=GenomeExp&file=index&option=ncbi&gsource=oral&org=smut&gprog=gview&geneid=SMU.112c) | SMU.112c transcriptional regulator; SIS_RpiR domain **^f^** |  | | |  | +3.0 |  |
| SMU.1517c | VicR; CheY-like receiver domain | -1.7 | | | -2.1^†^ |  |  |
| [SMU.1599](http://genome.brop.org/modules.php?op=modload&name=GenomeExp&file=index&option=ncbi&gsource=oral&org=smut&gprog=gview&geneid=SMU.1599) | CelR; GntR-like domain |  | | |  | +125.3 | +3.8^‡^ |
| SMU.1917 | ComE; LytTr DNA-binding domain (LytR/AlgR family) |  | | |  | +2.5 |  |
| [SMU.1920](http://genome.brop.org/modules.php?op=modload&name=GenomeExp&file=index&option=ncbi&gsource=oral&org=smut&gprog=gview&geneid=SMU.1920)c | PgdA; GTP-binding protein domain |  | | |  | +5.4 |  |
| [SMU.1923c](http://genome.brop.org/modules.php?op=modload&name=GenomeExp&file=index&option=ncbi&gsource=oral&org=smut&gprog=gview&geneid=SMU.1923c) | NrdR; NrdR-like domain |  | | |  | +4.4 |  |
| [SMU.1988c](http://genome.brop.org/modules.php?op=modload&name=GenomeExp&file=index&option=ncbi&gsource=oral&org=smut&gprog=gview&geneid=SMU.1988c) | SMU.1988c DNA binding protein; DUF1033-like domain |  | | |  | +2.7 | +1.5^‡^ |
| **Metabolism and synthesis/processing of antibiotics/bacteriocins** | |  | | |  |  |  |
| SMU.1339c* | BacD; Condensation and EntF domains ^g^ | -3.2 | | |  | -5.0 |  |
| [SMU.1340](http://genome.brop.org/modules.php?op=modload&name=GenomeExp&file=index&option=ncbi&gsource=oral&org=smut&gprog=gview&geneid=SMU.1340)* | bacA2 surfactin synthetase | -2.8 | | |  | -4.1 |  |
| SMU.1341c* | GrsA; Condensation and EntF domains ^g^ | -3.2 | | |  | -4.2 |  |
| SMU.1342c* | BacA1; Peptide synthase-like domain | -3.2 | | | -6.0^†^ | -4.5 |  |
| [SMU.1343c](http://genome.brop.org/modules.php?op=modload&name=GenomeExp&file=index&option=ncbi&gsource=oral&org=smut&gprog=gview&geneid=SMU.1343c)* | putative polyketide synthase | -2.2 | | |  | -3.0 |  |
| SMU.1344c* | malonyl-CoA acyl-carrier; Acyl transferase domain | -3.2 | | | -7.4^†^ | -3.5 |  |
| [SMU.1345c](http://genome.brop.org/modules.php?op=modload&name=GenomeExp&file=index&option=ncbi&gsource=oral&org=smut&gprog=gview&geneid=SMU.1345c)* | putative peptide synthetase similar to MycA | -2.6 | | |  | -3.8 |  |
| SMU.1346c* | BacT; hydrolase domain | -3.2 | | | -4.6^†^ | -3.5 |  |
| SMU.1489 | LacX aldose 1-epimerase; Mutarotase-like domain | -5.0 | | | -1.8^‡^ |  |  |
| SMU.1881c | SMU.1881c; P-loop NTPase domain ^h^ | -3.6 | | | -1.8^‡^ |  |  |
| [SMU.1882c](http://genome.brop.org/modules.php?op=modload&name=GenomeExp&file=index&option=ncbi&gsource=oral&org=smut&gprog=gview&geneid=SMU.1882c) | hypothetical protein | -1.9 | | |  | -3.1 |  |
| SMU.1896c | BlpU; Bacteriocin_IIc domain | -33.2 | | | -18.9^†^ |  |  |

* indicates genes/operon-like gene clusters regulated by both CovR and VicR.

^a^ BSP-like repeat found in BSP/proteins which might control cell morphology in group B *Streptococcus*.

^b^ Present in protein for cell division (FtsE) and macrolide efflux carrier (MacAB).

^c^ Putative function in envelope biogenesis (lipid synthesis or cell division).

^d^ Degrade murein via cleavage of the beta-1,4-glycosidic bond between N-acetylmuramic acid and N-acetylglucosamine.

^e^ Present in conjugative transfer proteins.

^f^ Found in phosphosugar isomerases and phosphosugar binding proteins.

^g^ Found in multi-domain enzymes which synthesize peptide antibiotics.

^h^ Present in bacteriocin-processing endopeptidases.

^†^ p<0.01, ANOVA with *post hoc* Dunnett’s test.

^‡^ p<0.05, ANOVA with *post hoc* Dunnett’s test.

NS: Not significant
