# Supplementary material for: CovR and VicRK Regulate Cell Surface Biogenesis Genes Required for Biofilm Formation in Streptococcus mutans
Source: PLoS One. 2013 Mar 12;8(3):e58271. doi: 10.1371/journal.pone.0058271 (PMC3595261; doi:10.1371/journal.pone.0058271)
Supplement: Table S3 — Additional genes significantly down-regulated in UAcov. (cut-off 2.5 fold). (DOC) [file pone.0058271.s007.doc]

**Table S3. Additional genes significantly down-regulated in UAcov (cut-off 2.5 fold).**

| Locus ^a^ | NCBI description ^b^ | Fold ^c^ | P value ^d^ | Probability of differential expression ^d^ |
| --- | --- | --- | --- | --- |
| [SMU.41](http://genome.brop.org/modules.php?op=modload&name=GenomeExp&file=index&option=ncbi&gsource=oral&org=smut&gprog=gview&geneid=SMU.41) | hypothetical protein | -26.5 | 0.002 | 75.1% |
| [SMU.40](http://genome.brop.org/modules.php?op=modload&name=GenomeExp&file=index&option=ncbi&gsource=oral&org=smut&gprog=gview&geneid=SMU.40) | hypothetical protein | -15.2 | 0.000 | 100.0% |
| [SMU.208c](http://genome.brop.org/modules.php?op=modload&name=GenomeExp&file=index&option=ncbi&gsource=oral&org=smut&gprog=gview&geneid=SMU.208c) | putative transposon protein | -2.5 | 0.000 | 100.0% |
| [SMU.213c](http://genome.brop.org/modules.php?op=modload&name=GenomeExp&file=index&option=ncbi&gsource=oral&org=smut&gprog=gview&geneid=SMU.213c) | hypothetical protein | -2.5 | 0.002 | 80.0% |
| [SMU.644](http://genome.brop.org/modules.php?op=modload&name=GenomeExp&file=index&option=ncbi&gsource=oral&org=smut&gprog=gview&geneid=SMU.644) | putative competence protein/transcription factor | -7.3 | 0.003 | 68.5% |
| [SMU.1337c](http://genome.brop.org/modules.php?op=modload&name=GenomeExp&file=index&option=ncbi&gsource=oral&org=smut&gprog=gview&geneid=SMU.1337c) | putative alpha/beta superfamily hydrolase | -3.1 | 0.000 | 100.0% |
| [SMU.1363c](http://genome.brop.org/modules.php?op=modload&name=GenomeExp&file=index&option=ncbi&gsource=oral&org=smut&gprog=gview&geneid=SMU.1363c) | putative transposase | -2.5 | 0.000 | 100.0% |
| [SMU.1365c](http://genome.brop.org/modules.php?op=modload&name=GenomeExp&file=index&option=ncbi&gsource=oral&org=smut&gprog=gview&geneid=SMU.1365c) | putative permease | -2.6 | 0.000 | 100.0% |
| [SMU.1366c](http://genome.brop.org/modules.php?op=modload&name=GenomeExp&file=index&option=ncbi&gsource=oral&org=smut&gprog=gview&geneid=SMU.1366c) | ABC transporter; ATP-binding protein | -2.5 | 0.000 | 99.9% |
| [SMU.1367c](http://genome.brop.org/modules.php?op=modload&name=GenomeExp&file=index&option=ncbi&gsource=oral&org=smut&gprog=gview&geneid=SMU.1367c) | hypothetical protein | -2.5 | 0.000 | 100.0% |
| [SMU.1837](http://genome.brop.org/modules.php?op=modload&name=GenomeExp&file=index&option=ncbi&gsource=oral&org=smut&gprog=gview&geneid=SMU.1837) | aroH phospho-2-dehydro-3-deoxyheptonate aldolase | -2.5 | 0.000 | 100.0% |

^a^ SMU numbers designate open reading frames based on *S. mutans* UA159 genome annotation.

^b^ Gene description based on nomenclature used NCBI database nomenclature (<http://www.ncbi.nlm.nih.gov/gene>) (April, 2012).

^c^ Change in the transcripts levels of the *covR* mutant (UAcov strain) compared to the wild type UA159 (∆*covR*/WT).

^d^ Analysis was performed using the LIMMA algorithm interface available online at www.brop.org.
